# Stop What You're Doing!—An fMRI Study on Comparisons of Neural Subprocesses of Response Inhibition in ADHD and Alcohol Use Disorder

**DOI:** 10.3389/fpsyt.2021.691930

**Published:** 2021-09-16

**Authors:** Sarah Gerhardt, Mathias Luderer, Jan M. Bumb, Esther Sobanski, Franz Moggi, Falk Kiefer, Sabine Vollstädt-Klein

**Affiliations:** ^1^Department of Addictive Behavior and Addiction Medicine, Central Institute of Mental Health, Medical Faculty Mannheim, University of Heidelberg, Mannheim, Germany; ^2^Department of Psychiatry, Psychosomatic Medicine and Psychotherapy, University Hospital, Goethe University Frankfurt, Frankfurt, Germany; ^3^Department of Psychiatry and Psychotherapy, Central Institute of Mental Health, Medical Faculty Mannheim, University of Heidelberg, Mannheim, Germany; ^4^Department of Child and Adolescent Psychiatry, University Medical Center Mainz, Mainz, Germany; ^5^Translational Research Center, University Hospital of Psychiatry and Psychotherapy, University of Bern, Bern, Switzerland; ^6^Mannheim Center for Translational Neurosciences, Medical Faculty Mannheim, University of Heidelberg, Mannheim, Germany; ^7^Feuerlein Center on Translational Addiction Medicine, University of Heidelberg, Heidelberg, Germany

**Keywords:** ADHD, alcohol use disorder, response inhibition, inhibitory control, fMRI, impulsivity

## Abstract

**Rationale:** Both attention deficit-/hyperactivity disorder (ADHD) and alcohol use disorder (AUD) are accompanied by deficits in response inhibition. Furthermore, the prevalence of comorbidity of ADHD and AUD is high. However, there is a lack of research on whether the same neuronal subprocesses of inhibition (i.e., interference inhibition, action withholding and action cancellation) exhibit deficits in both psychiatric disorders.

**Methods:** We examined these three neural subprocesses of response inhibition in patient groups and healthy controls: non-medicated individuals with ADHD (ADHD; *N* = 16), recently detoxified and abstinent individuals with alcohol use disorder (AUD; *N* = 15), and healthy controls (HC; *N* = 15). A hybrid response inhibition task covering interference inhibition, action withholding, and action cancellation was applied using a 3T functional magnetic resonance imaging (fMRI).

**Results:** Individuals with ADHD showed an overall stronger hypoactivation in attention related brain areas compared to AUD or HC during action withholding. Further, this hypoactivation was more accentuated during action cancellation. Individuals with AUD recruited a broader network, including the striatum, compared to HC during action withholding. During action cancellation, however, they showed hypoactivation in motor regions. Additionally, specific neural activation profiles regarding group and subprocess became apparent.

**Conclusions:** Even though deficits in response inhibition are related to both ADHD and AUD, neural activation and recruited networks during response inhibition differ regarding both neuronal subprocesses and examined groups. While a replication of this study is needed in a larger sample, the results suggest that tasks have to be carefully selected when examining neural activation patterns of response inhibition either in research on various psychiatric disorders or transdiagnostic questions.

## Introduction

Have you ever been drinking more than intended, or answered hastily to a question without listening until the end? *Impulsivity* can be seen as a failure to withhold or stop a response while being aware of negative consequences (1, 2). It therefore also modulates *response inhibition* (3) which, in turn, can be considered when one plans to operationalize impulsivity. Within the field of mental disorders, impulsive behavior, in particular, failed response inhibition, plays a major role and is reflected in the diagnostic criteria of attention deficit-/hyperactivity disorder (ADHD) and alcohol use disorder (AUD) (4).

ADHD and AUD have great impact on a person's life, her or his social environment, and the health care system. The global prevalence of ADHD is estimated to be 3.4% in children and adolescents (5) and between 2.5 and 4.4% in adults (6, 7). Regarding AUD, 12-months-prevalence rates of 11.0% for alcohol abuse, and 3.6% for alcohol dependence were reported for Germany (8) and lifetime prevalence of AUD and severe AUD were 29.1 and 13.9%, respectively, for the USA (9). ADHD and substance use disorder (SUD), including AUD, are often reported as co-occurring disorders (10, 11) with a prevalence of about 23% in SUD treatment seeking patients (12). Luderer et al. (13) reported a prevalence of ADHD of 20.5% in AUD patients in a German long-term inpatient setting. Children with ADHD have a significantly higher risk of developing an SUD (14), so do adolescents (15). ADHD also leads to a weaker treatment efficacy and higher relapse rates in AUD (13, 16) and a more severe course of illness in SUD (17, 18). Failure in response inhibition is considered as one of the underlying mechanisms in both ADHD and AUD (19–22), possibly being a link between both disorders (23). Underlying neurobiological deficits, e.g., dopaminergic deficits or a fronto-striatal hypofunction, also play a role in the co-occurrence of ADHD and SUD (24–26). Additionally, an increased risk for binge drinking in adolescence due to ADHD collides with critical phases regarding brain maturation. This aggravates impairments in response inhibition and increases, in turn, the risk for further binge drinking episodes and possibly a subsequent SUD (27).

In general, response inhibition is related to brain activation in frontal and parietal, and subcortical regions such as the thalamus and basal ganglia (3, 28–30). An hybrid response inhibition (HRI) task can be used to concurrently examine the three subprocesses of response inhibition (31), namely interference inhibition, action withholding, and action cancellation, by combining a Simon, a Go-/No-Go-, and a Stop-Signal task. In a functional magnetic resonance imaging (fMRI) study, all subtasks led to activation in key regions of response inhibition. Pre-motor and parietal regions were observed to be more active during interference inhibition. Action withholding showed overlapping regions with interference inhibition and action cancellation. Fronto-striatal regions were more active during action cancellation, thus, with increased stopping demands (31). In a recent meta-analysis, Zhang et al. (32) observed engagement of a fronto-parietal and ventral attention network during all subprocesses. However, differentiating between subprocesses, stronger activation in the ventral attention network for interference inhibition was observed. Further, action withholding, and action cancellation led to a stronger activation in the fronto-parietal network. These subprocesses follow the concept by Barkley (2): (1) inhibition of the initial response to an event where the response had previously been associated with any sort of reinforcement; (2) cancellation of the already ongoing response which includes a delay in the decision to respond; and (3) protection of a response over a period of delay due to competing events and responses.

Both, ADHD and AUD show alterations in brain activation during response inhibition compared to healthy controls (HC). In ADHD, impaired response inhibition has been observed in all three subprocesses. Hypoactivation during a Go-/No-Go task was observed in frontal, parietal and subcortical areas (33). While performing a Stop-Signal task, hypoactivation in inferior frontal, motor and subcortical areas (34) became apparent. Contrasting the three subprocesses of response inhibition, individuals with ADHD showed hypoactivation in parietal and frontal areas during interference inhibition, and hypoactivation in frontal and striatal areas during action withholding and action cancellation (35). Meta-analyses observed hypoactivation in frontal and subcortical areas (26), and additionally in motor and temporal areas (36). Hyperactivation in visual and dorsal attention networks, e.g., in frontal and parietal areas, may compensate for deficits (37). Deficits in response inhibition were also linked to dysfunctions in lateral prefrontal regions and the anterior cingulate cortex, whereas a hypofunction in the fronto-striatal network occurs during successful behavioral inhibition (38).

In AUD, subprocesses of response inhibition are impaired as well. While performing a Go-/No-Go task, hypoactivation in frontal, parietal and subcortical areas were observed (39, 40). In heavy drinkers compared to light drinkers, hypoactivation was observed in motor, parietal, frontal, temporal and subcortical regions (41). During the Stop-Signal-Task, hyperactivation in subcortical areas and hypoactivation in motor regions were found in AUD (42). Systematically reviewing recent findings on deficits in response inhibition in SUD, three quarter of the studies observed hypoactivation within the salience, executive and memory network (43).

The current study aims to identify differences in clinical populations (ADHD and AUD) and compared to healthy controls (HC) regarding task-related neural activation during three subcomponents of response inhibition using the HRI task. (1) We expected a poorer behavioral performance in individuals with AUD or ADHD in contrast to HC. (2) We aimed to replicate previous findings in HC on common but also distinct neural activation patterns for all three subprocesses. (3) A differential neural response, such as hypoactivation in frontal, parietal or subcortical brain regions, in patients (AUD and ADHD) compared to HC was expected for (3a) all subprocesses of response inhibition but (3b) to be more pronounced for action withholding and action cancellation. (4) Comparing individuals with AUD to those with ADHD, we expected compensatory regulation *via* increased recruitment of brain regions (4a) in subcortical regions for AUD, and (4b) in visual and dorsal attention regions for ADHD.

## Materials and Methods

### Participants

From October 2014 to June 2017, 57 individuals participated in this study. All patients were recruited at the Central Institute of Mental Health (Mannheim, Germany). Healthy controls answered to public announcements or were contacted using a list containing participants from previous studies that gave written consent about being contacted for further studies. In accordance with the Declaration of Helsinki, written informed consent was provided by all participants. The Ethics Committee of the University of Heidelberg approved the study (No. 2013-530N-MA).

### Procedure

All volunteers were screened for study in- and exclusion criteria. Regardless of the later group allocation, all individuals were excluded for any mental disorders within 1 year prior to participation, such an anxiety, depressive disorders, or a lifetime diagnosis of delusional disorders, schizophrenia, or bipolar disorder irrespectively of their group allocation. A possible history of oppositional defiant disorder, conduct disorder and other diagnoses during childhood were not examined in our adult study sample. Intake of other psychotropic medication (also ADHD-related medication) led to an exclusion of the study. Current and steady use of selective serotonin reuptake inhibitors and the diagnosis of a mild depressive episode were tolerated for patients (AUD or ADHD). All volunteers underwent a questionnaire-based screening for ADHD and AUD. Depending on the suspected diagnosis, individuals followed a subsequent diagnostic procedure: Individuals with AUD were diagnosed by trained masters- or medical-degreed personnel of the Department of Addictive Behavior and Addiction Medicine according to the International Classification of Diseases [ICD-10; (44)]. Participants were abstinent for at least 5 days prior to study inclusion (mean 19.0 days) and successfully completed a medically supervised detoxification [CIWA-Ar < 7 (45)]. Individuals with ADHD were diagnosed by trained masters- or medical-degreed personnel of the Psychiatric Outpatient Clinic of the Department for Psychiatry and Psychotherapy according to clinical guidelines (English version of the German Guidelines for the diagnostic procedure of ADHD: https://www.awmf.org/leitlinien/detail/ll/028-045.html). Besides structured interviews, school records and informant's ratings were used for the diagnostic procedure, if available. Individuals with ADHD were included, if they were medication free (e.g., methylphenidate: no intake for at least 8 weeks) prior to study participation. A diagnosis of ADHD in the AUD group and vice versa was ruled out following the same procedure. After group allocation, sociodemographic information was collected, and all participants filled out ADHD- and AUD-related and other questionnaires and took part in the fMRI assessment. For questionnaires, in- and exclusion criteria, see Supplementary Material and Supplementary Table A.1.

### Task

**Figure 1:**
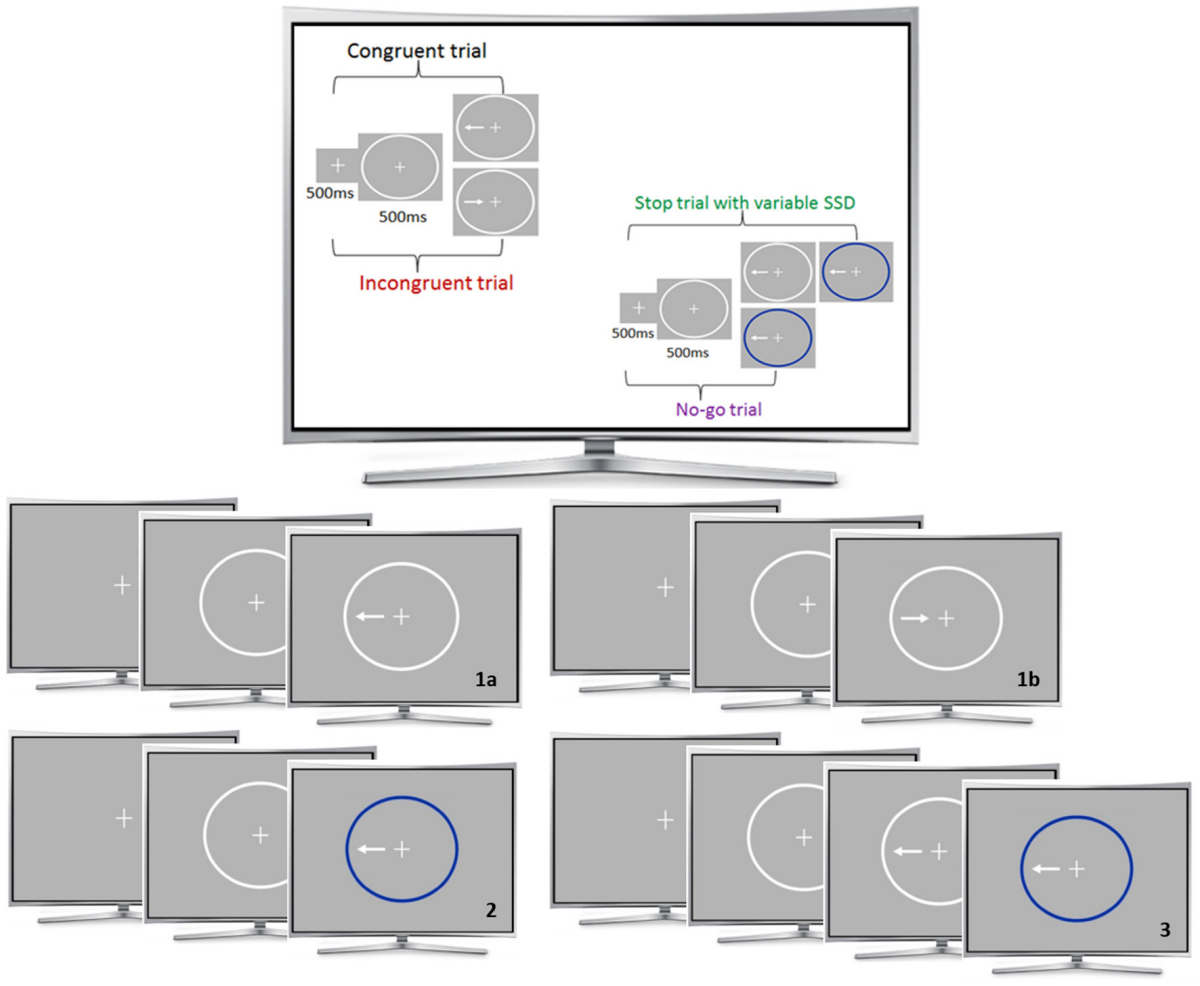
fMRI task. The Simon-task included congruent (1a) and incongruent (1b) trials. The Go-/No-Go (2) and Stop conditions (3) consisted of congruent trials. A fixation cross was presented for 500 ms at the beginning of each trial, followed by a white ellipse (500 ms) and the task condition. The stop-signal delay varied between 20 and 220 ms. During the 160 trials of each run, the four different stimuli conditions were presented in a pseudo-randomized order: a congruent go condition (62.5%), an incongruent go condition (12.5%), a no-go condition (12.5%) and a stop condition (12.5%).

The HRI fMRI task implemented in this study was already used and validated before (31). The combined task consists of Simon-, Go-/No-Go- and Stop-Signal trials that were integrated into one paradigm to assess interference inhibition, action withholding and action cancellation (Figure 1). A detailed description of the task can be found in the Supplementary Material.

### Functional MRI Acquisition

Functional MRI data was acquired using a 3T whole-body tomograph (MAGNETOM Trio, TIM-technology; Siemens, Erlangen, Germany). For the HRI task, 217 T2^*^-weighted echo-planar images were acquired per run in 8:48 min (TR = 2.41 s, TE = 25 ms, flip angle = 80°, 42 slices, slice thickness: 2 mm, 1 mm gap, voxel dimensions 3 × 3 × 3 mm^3^, FOV 192 × 192 mm^2^, 64 × 64 in-plane resolution). Additionally, a 5:21 min anatomical scan was performed to acquire a T1-weighted 3D MPRAGE dataset (192 sagittal slices, TR = 2.30 s, TE = 3.03 ms, TI = 900 ms, flip angle = 9°, slice thickness: 1 mm, 0.5 mm gap, voxel dimensions 1 × 1 × 1.5 mm^3^, FOV 256 × 256 mm^2^, 256 × 256 in-plane resolution). Images were presented to participants using MRI Audio/Video Systems goggles (Resonance Technology Inc., Los Angeles, CA, USA) with integrated lenses if necessary. Task presentation and data recording were realized using Presentation® software (Version 16.5, Neurobehavioral Systems, Inc., Albany, CA, USA).

### Functional MRI Pre-processing and Data Analysis

The fMRI data was preprocessed and analyzed using SPM8 (Statistical Parametric Mapping; Wellcome Trust Center for neuroimaging, London, UK). Field maps were used to control for magnetic field inhomogeneity. To ensure a steady state longitudinal magnetization, the first five volumes of each run were excluded. The remaining images (212 images for each HRI-run) were spatially realigned to the first image of the first HRI-run to correct for head motion over the course of the three runs. Further, slice-time correction was performed. The anatomical image was normalized to a MNI (Montreal Neurological Institute, Quebec, Canada) EPI template. Images were smoothed with an isotropic Gaussian kernel (8 mm full width at half maximum, FWHM). Images from all three runs were used for the following first- and subsequent second-level analyses, e.g., for creating contrasts according to our study aim.

Statistical analysis on the first level (single subject) was performed by fitting a linear regression model (general linear model, GLM) on a voxel-to-voxel basis. Realignment parameters were included as regressors of no interest. Stick functions at stimulus onset were used to model all events. Further, a convolution with the canonical hemodynamic response function was performed and a high-pass filter (cut-off at 128 s) was used. The following contrasts were used for further analyses: incongruent go > congruent go (interference inhibition), no-go > congruent go (action withholding) and stop > congruent go (action cancellation), correct reactions each. Additionally, the contrasts action withholding > interference inhibition, action cancellation > interference inhibition, action cancellation > action withholding were modeled. A quality check was performed, and all datasets were screened for successful preprocessing steps. Individuals with artifacts or excessive head movement (> 3 mm/3°) were excluded from the subsequent analysis. Please see Supplementary Table A.3 and “4. Head motion – calculation and group comparison” for additional information regarding motion parameters. No significant difference in head motion was observed between groups.

A one-sample *t*-test, including HC only, was performed for all three contrasts to assess the task effects. A full factorial model, including all three participant groups, was chosen for each contrast of interest separately, to test for group effects (interference inhibition, action withholding, and action cancellation). Regarding differences between subprocesses and groups, three additional full factorial models were estimated, using the contrasts action withholding > interference inhibition, action cancellation > interference inhibition, action cancellation > action withholding. On significant, anatomical region per contrast and group comparison was chosen and anatomical brain masks were created using the WFU_Pick_Atlas (anatomical automatic labeling) implemented in SPM. In order to demonstrate group differences eigenvariates were extracted within these masks and ANOVA and corresponding *post-hoc* tests were used to confirm significant group differences. Results are displayed using bar graphs. In order to examine brain-behavior relations, we performed a multiple regression analysis including the imaging data of the overall sample. Following the significant group differences on the behavioral level, the rate of commission errors was chosen as a covariate. Correspondingly, we used imaging data from the first level contrast for action withholding. Relevant frontal, motor and subcortical regions were identified and anatomical brain masks were created, again using the WFU_Pick_Atlas (anatomical automatic labeling) implemented in SPM. In order to demonstrate the relation between neural activation during action withholding and the rate of commission errors, eigenvariates from the first level results from all individuals were extracted within these masks. Results are displayed using scatter plots.

Age was included as a covariate in all analyses due to significant group differences. To control for multiple statistical testing, the probability for a family-wise error (FWE) was set to 0.05. Using 10,000 Monte Carlo Simulations and an automatic estimation of smoothness in AFNI's 3dClustSim (Analysis of Functional NeuroImages, www.afni.nimh.nih.gov/) a cluster-defining primary threshold (CDT; *P* < 0.01) in combination with a cluster-extend threshold of *k* ≥ 460 (group comparisons) or k ≥ 452 (HC only) was applied. The average smoothness in each direction was extracted from the SPM residuals using the individual SPM.mat files. A Gaussian-shaped spatial auto-correlation was modeled with estimated smoothness specified from each dimension (x, y, z) separately. The spatial resolution used for the GLM was 2 × 2 × 2. A whole brain mask as a union of the SPM templates “EPI.nii” and “brainmask.nii” was used. Results were visualized using MRIcro (46). Sample characteristics and questionnaires were analyzed using the Statistical Package for the Social Sciences (SPSS) version 25.0 (IBM Corporation, Armonk, NY, USA). Mean reaction time of correct go trials was calculated. Commission errors were defined as percentage of incorrect responses to all No-Go trials. Failure to stop was assessed as percentage of incorrect responses to all Stop trials. Subtracting the mean RT of the congruent trials from the mean RT of the incongruent trials resulted in the interference effect. The stop-signal reaction time was calculated by subtracting the average stop-signal delay from the median RT for correct reactions during go trials. One-way analyses of variance (ANOVA) or Welch-tests were chosen for group comparisons. For comparing patients (AUD, ADHD) and HC independent samples *t*-tests were performed.

## Results

*N* = 57 individuals participated in the study. *N* = 46 individuals were included in the fMRI analysis, consisting of 15 AUD, 16 ADHD, and 15 HC. AUD- and ADHD-related measures resulted in significant group differences (*P* < 0.05) ensuring the quality of our diagnostic and group allocation procedure. Groups did not differ in their number of days of abstinence (*P* > 0.05) and regarding gender, marital status, number of children, education, employment (*P* > 0.05). However, each group included more men than women. Individuals with ADHD were significantly younger than AUD [*F*_(2,43)_ = 6.56, *P* = 0.003; *N* = 46]. Sample characteristics (e.g., sociodemographic data and questionnaire scores) and corresponding statistics are displayed in Table 1. See CONSORT flow-chart in the Supplementary Material for inclusion and group allocation procedure.

**Table 1:** Group characteristics of all participants including corresponding statistics.

	**AUD (*N* = 15)**	**ADHD (*N* = 16)**	**HC (*N* = 15)**	**Pearson Chi-Square/ANOVA^a^/Welch^b^**
**Gender** (male:female)	13:2	12:4	9:6	χ^2^_(2)_ = 2.78, *p* = 0.249; (*N* = 46)
**Age** [years; mean (SD)]	47.0 (12.3)^1^	31.2 (10.4)^1^	41.9 (14.4)	*F*_(2,43)_ = 6.56, *p* = 0.003^a^; (*N* = 46)
**Smoker** (yes:no:unknown)	9:4:2	4:12:0	2:12:1	χ^2^_(2)_ = 10.06, *p* = 0.007; (*N* = 43)
**Marital status** (married:separated:single:unknown)	4:1:6:4	4:1:8:3	4:1:9:1	χ^2^_(4)_ = 0.25, *p* = 0.993; (*N* = 38)
**Children** (yes:no:unknown)	2:5:8	7:6:3	8:6:1	χ^2^_(2)_ = 1.45, *p* = 0.439; (*N* = 34)
**Education** (years; mean (SD)]	12.7 (2.3)	14.1 (1.9)	14.1 (3.1)	*F*_(2,35)_ = 1.186, *p* = 0.318^a^; (*N* = 38)
**Employment** (employed:unemployed:retired:unknown)	7:3:1:4	13:1:2	11:3:1:0	χ^2^_(6)_ = 8.73, *p* = 0.189; (*N* = 40)
**AUDIT** (mean (SD)]	27.1 (5.0)^1, 2^	3.2 (3.2)^1^	3.3 (2.3)^2^	*F*_(2,18.2)_ = 108.51, *p* < 0.000^b^; (*N* = 37)
**ADS** [mean (SD)]	16.1 (6.1)^1, 2^	3.0 (4.3)^1^	2.8 (3.0)^1^	*F*_(2,36)_ = 33.03, *p* < 0.000^a^; (*N* = 39)
**Days of abstinence** [mean (SD)]	19.0 (12.3)	49.5 (78.2)	13.1 (14.0)	*F*_(2,21.3)_ = 1.77, *p* = 0.109^b^; (*N* = 39)
**WURS-k** [mean (SD)]				
**Attention deficits/hyperactivity**	6.1 (5.7)^1^	16.9 (6.2)^1, 2^	5.5 (5.4)^2^	*F*_(2,36)_ = 16.95, *p* < 0.000^a^; (*N* = 39)
**Impulsivity**	1.8 (2.0)^§^	6.0 (4.9)^§%^	1.4 (1.8)^%^	*F*_(2,20.9)_ = 5.32, *p* = 0.014^b^; (*N* = 39)
**ADHD-SR** [mean (SD)]				
**Attention deficits**	2.7 (3.2)^1^	18.4 (4.1)^1, 2^	2.9 (2.6)^2^	*F*_(2,36)_ = 96.23, *p* < 0.000^a^; (*N* = 39)
**Hyperactivity**	2.2 (2.9)^1^	6.4 (4.9)^1, 2^	0.6 (0.8)^2^	*F*_(2,15.3)_ = 10.05, *p* = 0.002^b^; (*N* = 39)
**Impulsivity**	1.4 (1.6)^1^	6.2 (3.2)^1, 2^	0.8 (1.0)^2^	*F*_(2,18.4)_ = 17.56, *p* < 0.000^b^; (*N* = 39)
**Overall score**	6.3 (5.5)^1^	30.9 (7.5)^1, 2^	4.3 (3.5)^2^	*F*_(2,19.2)_ = 71.56, *p* < 0.000^b^; (*N* = 39)

*AUDIT, Alcohol Use Disorder Identification Test; ADS, Alcohol Dependence Scale; WURS-k, Wender-Utah-Rating-Scale; ADHS-SR, ADHD-Selfrating Scale; ^a/b^ANOVA^a^/Welch^b^ for group differences; Tukey or Games-Howell post-hoc tests^1,2^, P < 0.05*.

### Behavioral Data

Comparing AUD, ADHD, and HC, statistical analyses did not reveal significant group differences regarding reaction time, interference effect, stop-signal reaction time, and omission errors (*P* > 0.05; see Table 2). Group differences were observed regarding commission errors [*F*_(2,23.1)_ = 3.77, *P* = 0.038; see Table 2]; *post-hoc* tests did show significant differences between ADHD and HC. In direct comparison between ADHD and HC, ADHD showed a higher stop-signal reaction time [two sample *t*-Test; *t*_(29)_ = 2.05, *P* = 0.025]. No significant differences were observed regarding all variables for the comparison between patients (AUD, ADHD) and HC, besides patients exhibiting a higher stop-signal reaction time [*t*_(44)_ = 2.11, *P* = 0.040; see Supplementary Table A.2].

**Table 2:** Behavioral data of AUD, ADHD, and HC for the HRI-task.

	**AUD (*N* = 15)**	**ADHD (*N* = 16)**	**HC (*N* = 15)**	**ANOVA^a^/Welch^b^**
**Reaction time congruent trials** [ms; mean ± SD]	553 ± 108	490 ± 108	507 ± 105	*F*_(2,43)_ = 1.40, *p* = 0.257^a^
**Reaction time incongruent trials** [ms; mean ± SD]	643 ± 105	594 ± 111	609 ± 100	*F*_(2,43)_ = 0.87, *p* = 0.425^a^
**Commission errors (no-go)** (%; mean ± SD)	1.4 ± 2.2^1^	10.2 ± 14.6^1^	3.6 ± 4.6	***F***_**(2, 23.1)**_**=3.77**, ***p*****=0.038**^**b**^
**Omission errors (no-go)** (%; mean ± SD)	3.6 ± 6.8	2.4 ± 2.7	6.8 ± 16.6	*F*_(2,21.5)_ = 0.65, *p* = 0.531^b^
**Failure to stop** (%; mean ± SD)	41.1 ± 11.7	49.1 ± 13.0	42.3 ± 13.0	*F*_(2,43)_ = 1.81, *p* = 0.176^a^
**Interference effect** (ms; mean ± SD)	90 ± 74	103 ± 58	101 ± 48	*F*_(2,43)_ = 0.22, *p* = 0.807^a^
**Stop-signal reaction time** (ms; mean ± SD)	261 ± 52	277 ± 69^2^	228 ± 64^2^	***F***_**(2, 43)**_**=2.47**, ***p*****=0.097^**a**^^2^**

*ANOVA, one-way analysis of variance; ^a/b^ANOVA^a^/Welch^b^ for group differences*;

1 *post-hoc test, P < 0.05*;

2 *In direct comparison, ADHD had significantly higher stop-signal reaction time than HC (two sample t-Test t_(29)_ = 2.05, p = 0.025)*.

*Significant results are highlightes in bold characters*.

### fMRI Results

#### Replication of the HRI Task

**Figure 2:**
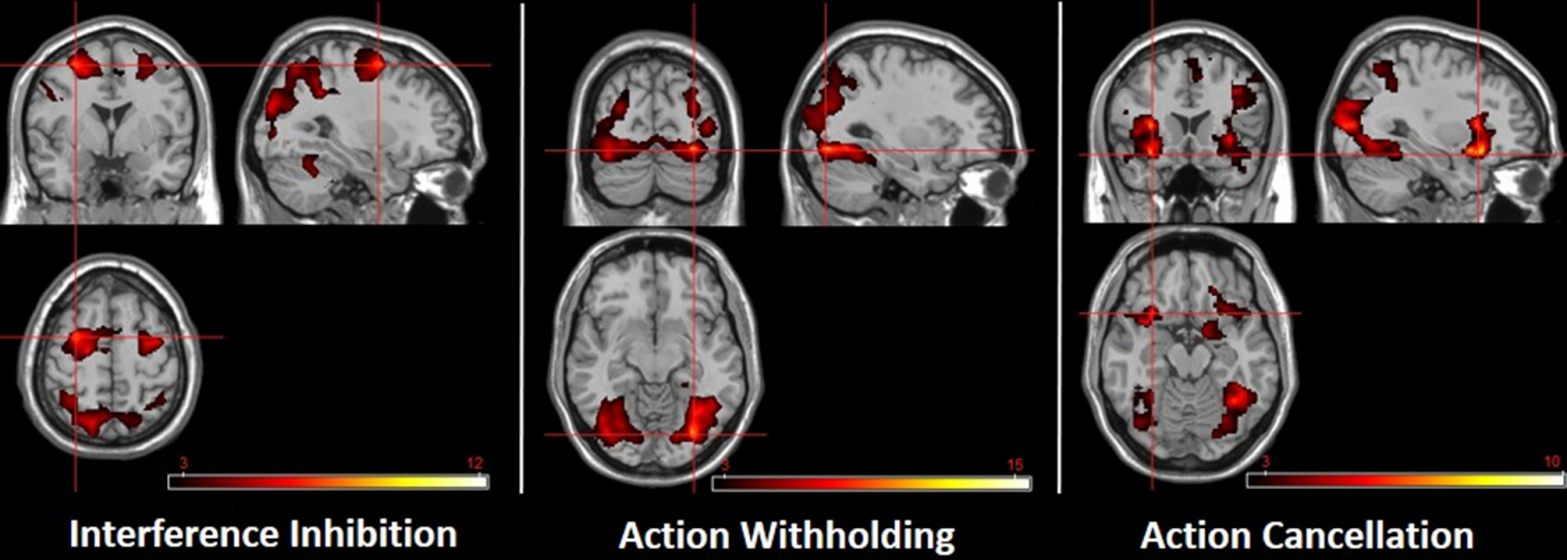
Subcomponents of response inhibition in healthy controls. The crosshairs highlight the location of the highest *t*-value. Left: interference inhibition (−30, 0, 58). Middle: action withholding (30, −78, −10). Right: action cancellation (−32, 18, −16). CDT of *P* < 0.01 (*k* ≥ 452). The color bar indicates t-scores, with red to yellow indicating a positive contrast.

In HC (*N* = 15), regions comparable to those reported by Sebastian et al. (31) survived the statistical threshold (CDT of *P* < 0.01, *k* ≥ 452). During interference inhibition, activation was observed in brain structures encompassing frontal (bilateral precentral, middle and superior frontal gyri, supplementary motor area, left inferior frontal gyrus), parietal (bilateral inferior, superior, post-central, supramarginal and angular gyri, precuneus), temporal (bilateral inferior and middle temporal lobe, fusiform gyrus) and occipital regions (bilateral inferior, middle and superior occipital gyrus, right cuneus). Action withholding led to activation in occipital (bilateral inferior, middle, superior, lingual gyri, calcarine, cuneus) and temporal regions (bilateral fusiform, and inferior temporal gyri, right middle temporal gyrus), as well as right parietal regions (superior parietal lobule, angular gyrus). During action cancellation, subcortical regions (bilateral insula, right putamen), as well as frontal (bilateral inferior frontal gyrus and supplementary motor area, right middle frontal, superior frontal and precentral gyri), parietal (bilateral inferior and superior parietal lobule, angular and supramarginal gyri, left precuneus), temporal (bilateral inferior, middle, superior, fusiform gyri) and occipital (bilateral inferior, middle, superior, lingual gyri, cuneus) regions were activated (Figure 2; Supplementary Table A.4). Following a rather explorative analysis (CDT of *P* < 0.05, *k* ≥ 1,497), further regions were involved. Additionally, the bilateral middle cingulate gyrus, putamen, hippocampus, insula and left amygdala and thalamus became apparent during interference inhibition. Action withholding led to additional activation in superior parietal and temporal regions. Moreover, action cancellation revealed additional activation in the anterior, middle and posterior cingulate gyrus, putamen, thalamus, hippocampus, amygdala, motor and parietal areas.

#### Group Comparison of Task-Related Functional Activation

##### Interference Inhibition (Simon-Task)

**Figure 3:**
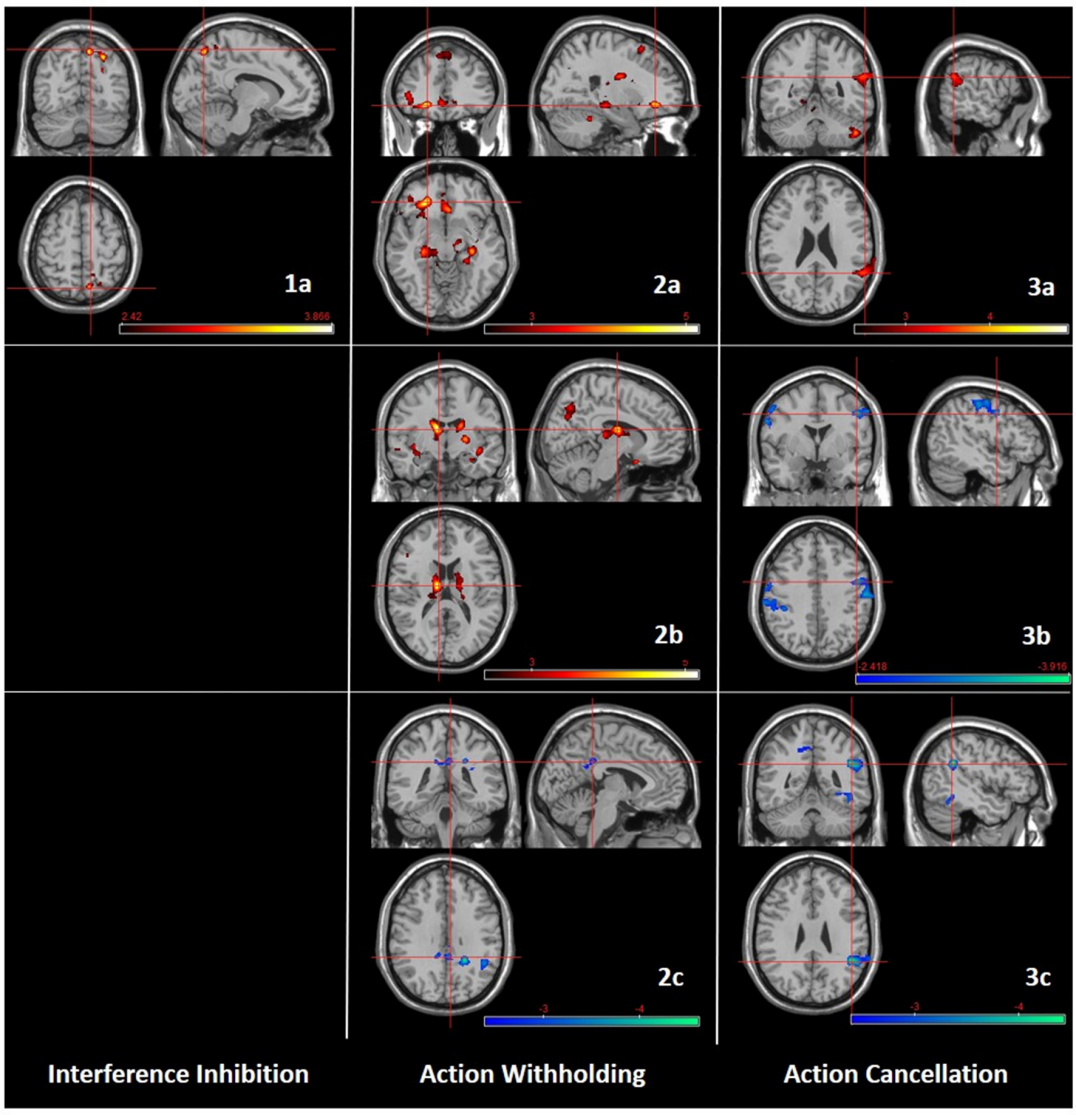
Group comparisons (AUD, ADHD, HC) regarding subcomponents of response inhibition. Left: Interference inhibition; 1a: AUD > ADHD (10, −68, 54). Middle: Action withholding; 2a: AUD > ADHD (−24, 36, −10); 2b: AUD > HC (−10, −10, 16); 2c: ADHD < HC (4, −40, 32). Right: Action cancellation; 3a: AUD > ADHD (58, −50, 24); 3b: AUD < HC (48, 0, 40); 3c: ADHD < HC (46, −48, 28). CDT of *P* < 0.01 (*k* ≥ 460). The color bar indicates *t*-scores, with red to yellow indicating a positive contrast and blue to green a negative contrast, respectively.

Individuals with AUD compared to ADHD showed more activation in the right superior parietal lobule, precuneus and superior and middle occipital gyri (Figure 3(1a); Supplementary Table A.5).

##### Action Withholding (Go-/No-Go Task)

Individuals with AUD compared to ADHD showed more activation in left frontal regions (inferior, middle, medial, and superior frontal gyri), left parietal regions (inferior parietal lobule, angular gyrus), left occipital regions (middle occipital gyrus), bilateral precuneus and fusiform gyrus, bilateral cingulate gyri (anterior, middle, posterior). In addition, individuals with AUD showed more activation in bilateral (hippocampus, parahippocampal gyrus, thalamus, caudate) and left (insula) subcortical structures compared to ADHD (Figure 3(2a); Supplementary Table A.6). Individuals with AUD compared to HC showed more activation in left frontal regions (inferior, middle and medial frontal gyri), left parietal regions (inferior parietal lobule, angular gyrus), mostly left temporal regions (temporal pole, middle and bilateral superior temporal gyri), left occipital regions (middle occipital gyrus, cuneus), and bilateral precuneus. In addition, individuals with AUD showed more activation in bilateral (insula, caudate, thalamus) and right (putamen, pallidum, hippocampus) subcortical structures compared to HC (Figure 3(2b); Supplementary Table A.6). Individuals with ADHD compared to HC showed less activation in bilateral cingulate gyri (posterior, middle) and right angular gyrus (Figure 3(2c); Supplementary Table A.6).

##### Action Cancellation (Stop-Signal Task)

Individuals with AUD compared to ADHD showed more activation in right parietal regions (angular and supramarginal gyri), temporal regions (left fusiform, right superior and middle temporal gyri) and left occipital regions (inferior occipital and lingual gyri) (Figure 3(3a); Supplementary Table A.7). Individuals with AUD compared to HC showed less activation in bilateral motor regions (precentral and post-central gyri), right superior and middle frontal gyri, left parietal regions (inferior parietal lobule, supramarginal gyrus) and left superior temporal gyrus (Figure 3(3b); Supplementary Table A.7). Individuals with ADHD compared to HC showed less activation in parietal regions (right angular and supramarginal gyri, left precuneus), right temporal regions (superior and inferior temporal gyri), right occipital regions (middle and superior occipital gyri) and bilateral fusiform, inferior occipital, and middle cingulate gyri (Figure 3(3c); Supplementary Table A.7).

##### Activation Profiles Regarding Subprocess and Group

**Figure 4:**
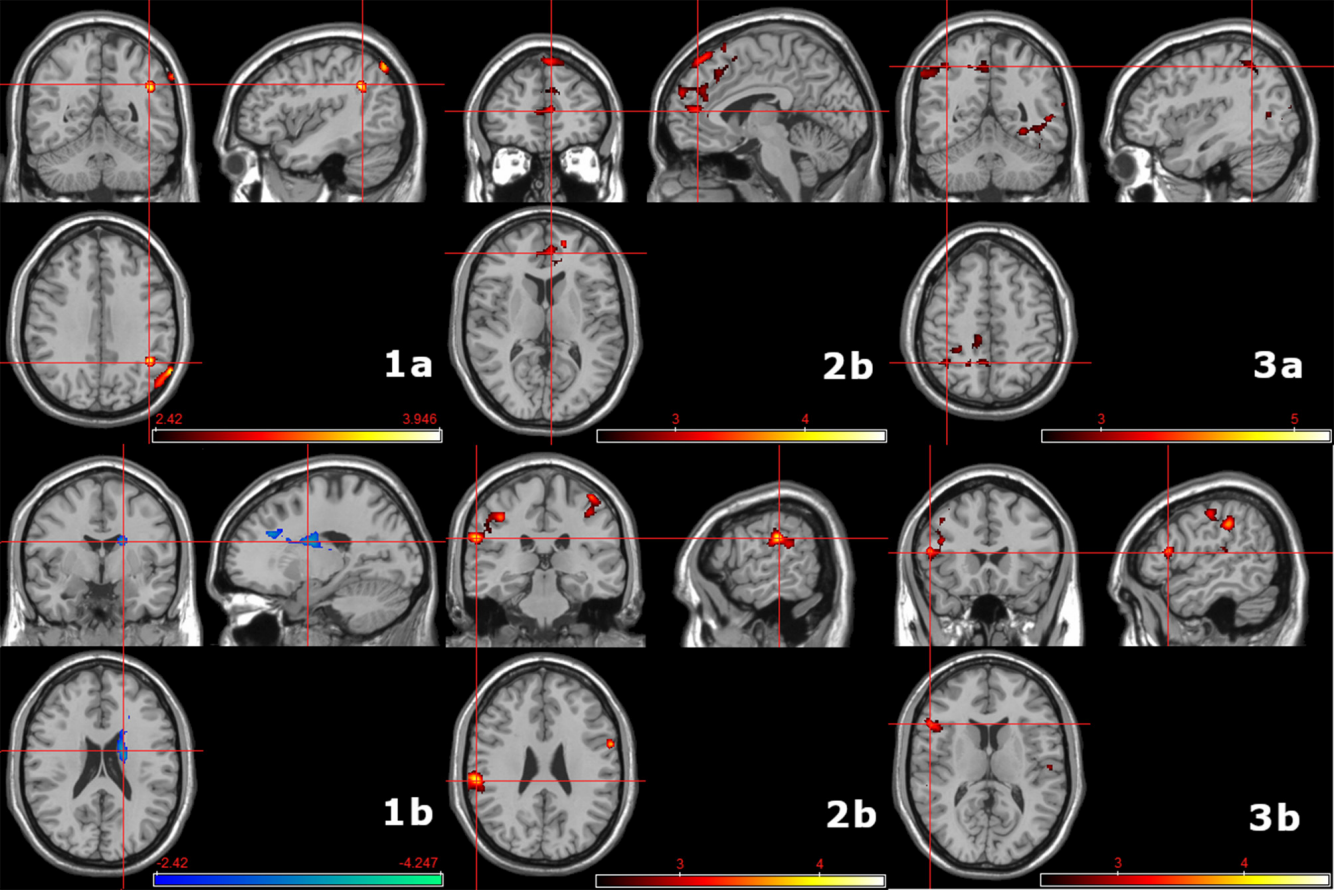
Group comparisons (AUD, ADHD, HC) regarding differences in subcomponents of response inhibition. Left: Action withholding > interference inhibition; 1a: HC > ADHD (44, −42, −32); 1b: HC < AUD (18, −2, 22). Middle: Action cancellation > interference inhibition; 2a: HC > ADHD (−24, 36, −10); 2b: HC > AUD (−10, −10, 16). Right: Action cancellation > action withholding; 3a: HC > ADHD (58, −50, 24); 3b: HC > AUD (48, 0, 40). See also Supplementary Figure A.1. CDT of *P* < 0.01 (*k* ≥ 460). The color bar indicates t-scores, with red to yellow indicating a positive contrast and blue to green a negative contrast, respectively.

Overall, significant group differences were observed between AUD or ADHD and healthy controls. No significant differences were observed between patient groups directy. Regarding action withholding > interference inhibition, individuals with ADHD exhibited less activation in the right angular gyrus (Figure 4(1a); Supplementary Table A.8) whereas individuals with AUD exhibited more activation within the right caudate, compared to HC (Figure 4(1b); Supplementary Table A.8). Contrasting action cancellation > interference inhibition, individuals with ADHD compared to HC exhibited less activation within superior frontal and anterior cingulate regions and the right inferior parietal gyrus (Figure 4(2a); Supplementary Table A.9). Individuals with AUD compared ton HC exhibited less activation mainly within left inferior parietal regions, bilateral motor regions and the right inferior frontal gyrus (Figure 4(2b); Supplementary Table A.9). Regarding action cancellation > action withholding, individuals with ADHD compared to HC exhibited less activation within occipital and bilateral temporal and parietal regions (Figure 4(3a); Supplementary Table A.10) whereas individuals with AUD showed less activation within left frontal and parietal regions, mostly left parietal regions and the bilateral insula (Figure 4(3b); Supplementary Table A.10). See also Supplementary Figure A.1 for additional bar plots displaying neural activation regarding before mentioned contrasts, comparisons and brain regions.

### Brain–Behavior Relation

**Figure 5:**
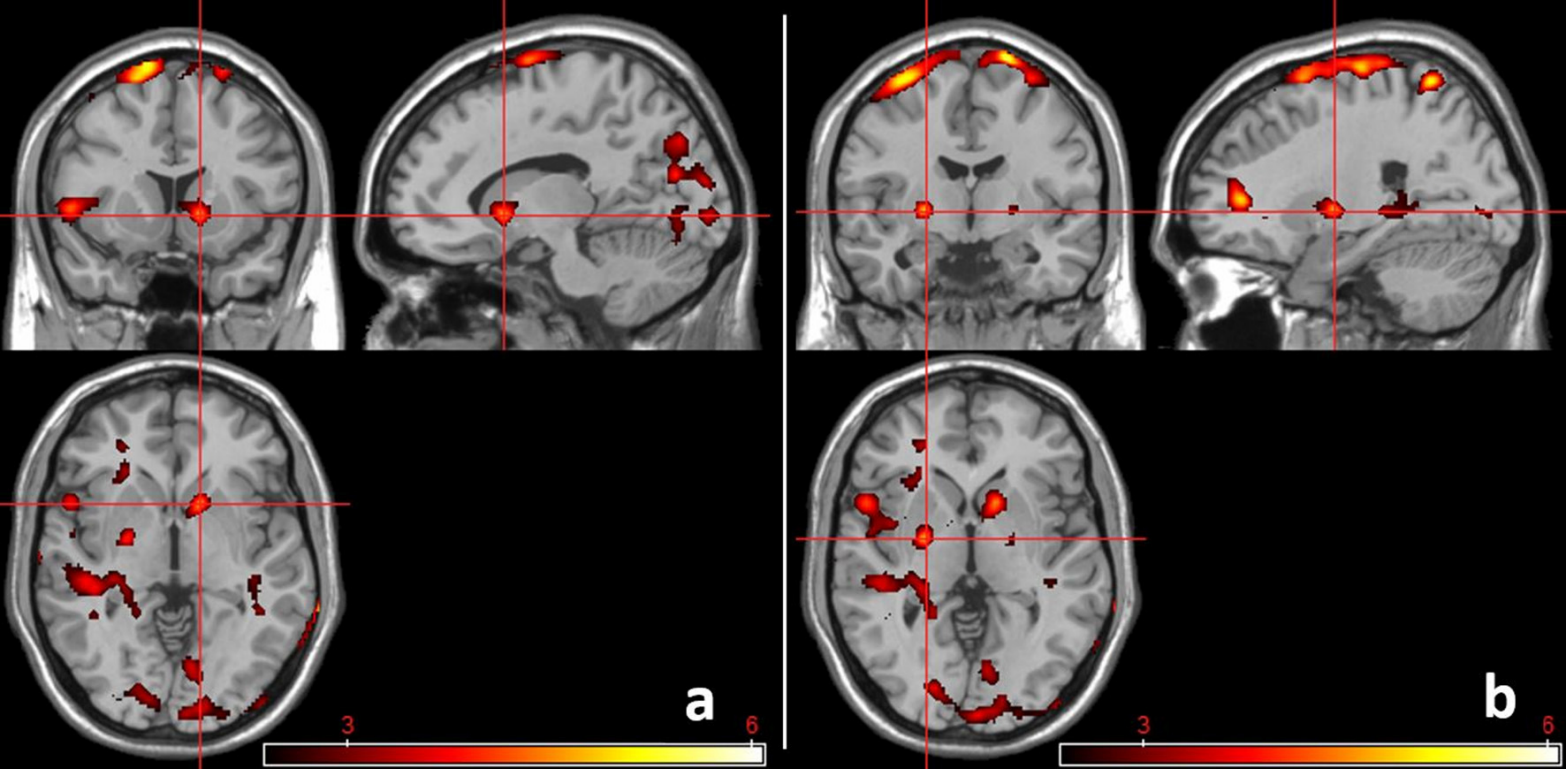
Brain activation during action withholding in positive relation to commission errors. All individuals were included in the regression analyses. Activation within motor regions and subcortical regions became apparent. **(a)** Activation within right subcortical regions (12, 12, −2). **(b)** Activation within left subcortical regions (−24, −6, 0). CDT of *P* < 0.01 (*k* ≥ 460). The color bar indicates t-scores, with red to yellow indicating a positive contrast.

A positive correlation was observed between neural activation during action withholding and the rate of commission errors. This was most prominent for bilateral motor regions (pre- and post-central gyri, supplementary motor area), bilateral subcortical regions (bilateral dorsal striatum, left insula and hippocampus), see Figure 5, Supplementary Figure A.2, and Supplementary Table A.11.

## Discussion

The current study examined neural subprocesses of response inhibition in individuals with AUD, ADHD and HC. Individuals with AUD show a high comorbidity with ADHD (12, 13). Since impaired response inhibition is seen as a common characteristic (19–22), a hybrid response inhibition task (31) was used in an fMRI experiment to assess different neural subprocesses of response inhibition. To the best of our knowledge, this is the first study directly comparing subprocesses of response inhibition and individuals with different mental disorders, namely AUD and ADHD, while also including a control group (HC).

### Behavioral Measures of Response Inhibition

Relating to hypothesis (1), patients exhibited significantly larger stop-signal reaction times than controls. Even though we did not find significant group differences (AUD, ADHD, and HC), it is worth noting that higher stop-signal reaction times were observed in ADHD in direct comparison to HC after excluding two outliers from the ADHD group. Individuals with ADHD also failed significantly more often in withholding a reaction to No-Go trial (commission error) compared to individuals with AUD. A higher rate of commission error and prolonged stop-signal reaction times with intact go reaction time have been observed previously in adults with ADHD (47), and are also considered as deficits in inhibitory control in individuals with substance use disorder (48). While some imaging studies did not observe significant group differences on the behavioral level [e.g., (39, 40)], others reported a correlation between stop-signal reaction times and stopping an initiated response, and binge drinking behavior (49). It has also been suggested that deficits in task performance in individuals with AUD, e.g., an increase in omission errors and no change in reaction times (48), might not only be due to deficits in response inhibition but also due to problems related to discrimination (50).

Whereas, others did not observe a relation between commission errors and neural reactivity in heavy drinkers (41), a higher rate of commission errors was associated with stronger activation in bilateral motor and subcortical regions during action withholding in the here present, overall sample. Individuals with higher activation in prefrontal and subcortical regions related to cognitive control and motor abilities exhibit impairments on the behavioral level. This hyperactivity during withholding a prepotent response might indicate a neural phenotype with regard to response inhibition. However, and with respect action withholding, a debate regarding neural correlates of efficiency and monitoring of cognitive control in contrast to inhibitory control *per se* is currently underway (51).

### Neural Subcomponents of Response Inhibition in Healthy Individuals

Regarding hypothesis (1), we observed a shift in activation from motor to visual to striatal areas regarding interference inhibition, action withholding and action cancellation. *Interference inhibition* led to a stronger activation mainly in motor, frontal and parietal regions. Activations observed here support the idea of the involvement of mostly attention-related networks during interference inhibition (32). Regarding *action withholding*, activation was observed in bilateral occipital and temporal regions as well as in parietal regions. *Action cancellation* led to extensive activation in fronto-parietal and striatal regions as well as in temporal and occipital areas. Both observations mostly match previous findings (32). Further regions were observed with a more explorative CDT. Differences on observed activation might be due to task categorization or task design. In their meta-analysis, the authors categorized several different tasks (e.g., Simon and Stroop task) as “interference inhibition” (32). In addition, Go-/No-Go-tasks and Stop-Signal tasks with different modalities (e.g., auditory) or difficulties were included. Further, our small sample size [*N*(HC) = 15] might contribute to not observing the exact same regions as reported previously. In direct comparison to a study using the same combined task (31), our results match previous findings: Interference inhibition results in activation of rather frontal, parietal and motor-related regions; action withholding leads to activation in visual and parietal regions; during action cancellation, inferior frontal, parietal, insular, striatal, and motor regions were active (31). Overall, this advocates further application of the task regarding the assessment of subcomponents of response inhibition in different clinical populations compared to healthy controls.

### Neural Subcomponents of Response Inhibition in ADHD and AUD Compared to HC

Contrary to our hypothesis (3a), we did not observe differences between patient groups and healthy individuals regarding all subprocesses of response inhibition. *Interference inhibition* did not lead to significant differences between ADHD or AUD and HC, although previous studies reported hypoactivation in networks related to attention, such as the anterior cingulate cortex, sensory and parietal regions (35), for ADHD compared to HC. In a different paradigm, our workgroup also did not observe neural alterations during interference inhibition in AUD compared to ADHD or HC, when considering only the neutral condition (52).

However, regarding (3b), we detected differences in neural activation during action withholding and action cancellation when comparing patient groups to HC. With increasing demand in response inhibition, differences between groups and subprocesses became more apparent. Firstly, this might underline a differentiation between interference inhibition and motor inhibition (action withholding and action cancellation) *per se*. Secondly, compared to HC, differences in neural activation were observable not only regarding patient groups and made them distinguishable in their neural activation, but also regarding action withholding and action cancellation.

Alterations in neural activation in ADHD compared to HC are represented as hypoactivation, mostly in cingulate and parietal regions during action withholding, and are furthermore widespread during action cancellation. Regarding *action withholding* in ADHD compared to HC, hypoactivation in parietal regions while successfully withholding a response has been reported previously for individuals with ADHD (33). However, others reported hypoactivation in parietal and cingulate regions (26, 35) during interference inhibition rather than during motor inhibition. Considering the similarity of the included population and procedure (e.g., diagnostic procedure and task) to the Sebastian et al. (35) study, these different findings were somewhat surprising. Being part of the default mode network, hypoactivation in both the angular and posterior cingulate gyri might indicate a compensatory mechanism, e.g., stronger redirection of attention, as activation of regions of the default mode network is known to be a task-negative mechanism (53). A positive correlation between stop-signal reaction times and default mode network (DMN) activation has been interpreted as impaired response inhibition—with the DMN interfering with task-specific attention redirection (54). In our sample, successfully withholding a response might lead to a stronger deactivation in the DMN in order to allow adequate response inhibition. When it comes to *action cancellation*, others reported reduced activation in inferior frontal, premotor, subcortical, cingulate regions (34, 35), indicating a dysfunction of the fronto-striatal network. However, we again observed a deactivation of DMN-related regions (mainly cingulate and precuneus) but also strong hypoactivation within the temporo-parietal junction (TPJ) and temporal and occipital regions. Hypoactivation in parieto-occipital but also temporal regions has been observed here but also previously regarding inhibition tasks (37). This can be seen as a failure to maintain response inhibition related processes, possibly leading to the prolonged stop-signal reaction time on a behavioral level. The TPJ is one of the key nodes in ventral attention (55) and response inhibition networks (56) and has been related to response inhibition previously (57). Even though we still observed hypoactivation in DMN-related regions, it is possible, that individuals with ADHD were not able to compensate during the more demanding Stop-Signal task.

Alterations in AUD compared to HC, however, are marked as hyperactivation in several clusters, encompassing frontal, temporal, parietal, occipital and subcortical regions during action withholding, and hypoactivation in bilateral motor-related regions during action cancellation. *Action withholding* led to hyperactivation in several clusters, encompassing frontal, temporal, parietal, occipital and subcortical regions. Reduced activation in frontal and parietal regions, as well as in the insula and anterior cingulate gyrus was related to AUD severity (40). Decreased activity in widespread brain regions has been observed previously in heavy drinkers compared to light social drinkers (41). This might contradict our results at first sight. However, Ahmadi et al. (41) compared college students (heavy vs. light drinkers)—a study sample that might display a different form of motivation toward abstinence. In addition, hypoactivation in left post-central and parietal regions has been observed in individuals with AUD compared to HC (39). In previous studies, mostly contextual action withholding tasks were administered. When comparing light to heavy social drinkers, contextual or neutral Go-/No-Go stimuli did not yield group differences in brain activation, indicating sustained response inhibition in these populations (58). In line with our observations, hyperactivation in motor, superior frontal and parietal regions, and the hippocampus was observed in drinkers compared to controls in a study by Hatchard et al. (59). Further, Stein et al. (60) observed hyperactivation in an fronto-striatal network in individuals with AUD compared to HC as well. The widespread hyperactivation observed in our AUD sample might indicate a compensatory mechanism for the response inhibition deficit leading to successful performance of the Go-/No-Go task in our paradigm. Hyperactivation in frontal and striatal regions, support the idea of a compensatory activation since both regions are known to be involved in response inhibition processes (25). Further, frontal and parietal regions contribute to attentional monitoring and salience attribution—processes that are also relevant during response inhibition (25). With demanding load on processes related to attention and response inhibition, brain structures such as occipital or subcortical regions are often involved in AUD, or other SUD (39). A reorganization of brain regions involved in attention and working memory has been discussed regarding AUD (61). Hyperactivation in AUD compared to HC might indicate either an alternative strategy to successfully perform a task or “replacing” previously responsible regions due to alcohol related impairments (61). During *action cancellation*, however, individuals with AUD exhibited hypoactivation in mainly motor-related regions. Interestingly, this was observed previously during an action withholding task (39). Others found hyperactivation of the right thalamus and hypoactivation of the left supplementary motor area in AUD compared to HC during a Stop-Signal task (42). We also did not observe hypoactivation of the left dorsolateral prefrontal cortex, as reported by others (62). The here observed stronger deactivation of motor-related regions might have helped maintaining performance on a behavioral level during the Stop-Signal task. In healthy individuals, a suppression of motor related activation was related to increased striatal activation during successful stopping of an ongoing response (63).

Our results indicate rather attention related alterations in individuals with ADHD and rather motor control related alterations in individuals with AUD compared to HC, respectively. The presence of neural group differences might imply alterations seen either as impairments (when leading to behavioral deficits) or as compensatory mechanisms (when maintaining an adequate level of performance). However, and possibly due to lack of power following the small sample size, we might have missed to observe a subcortical neural compensation or regulation of motor region activity. Also, facing contradictory results and with respect to differences in task administration, further studies are urgently needed.

Relating to hypothesis (4), we observed either compensatory neural activation or a shift in strategy for both ADHD and AUD. Alternative strategies, e.g., more strongly downregulation of DMN-related brain regions in individuals with ADHD, and upregulation of striatal or motor-related regions in individuals with AUD might lead to similar outcomes compared to HC on the behavioral level. Regarding hypothesis (4a), individuals with AUD showed hyperactivation in striatal, and further subcortical regions compared to HC during action withholding, and hypoactivation of motor regions during action cancellation. Individuals with ADHD, (4b), seem to compensate rather by more strongly downregulating regions of the DMN during action withholding, and possibly show deficits during action cancellation, which is represented by a prolonged stop-signal reaction times, compared to HC. This partly opposes the observation by Cortese et al. (37), describing a compensatory hyperactivation in visual and dorsal attention but also default mode networks during higher level cognitive tasks. However, since the meta-analysis by Cortese et al. (37) included different tasks addressing higher cognitive functions we cannot directly integrate our results into these observations.

Directly contrasting AUD and ADHD, healthy controls are needed to ascertain if differences in neural activation derive from hypo- or hyperactivation in individuals with AUD or ADHD, respectively.

### Neural Subcomponents of Response Inhibition in ADHD Compared to AUD

During *interference inhibition*, we observed stronger activation when contrasting AUD against ADHD in superior parietal regions. Previously, hypoactivation in parietal regions in individuals with ADHD have been observed during interference inhibition, supporting the hypothesis of a malfunctioning of selective attention networks. Consequently, this leads to impaired response selection—an early subprocess of response inhibition (35). The superior parietal lobule, including the precuneus, has been related to attentional processes, e.g., the shift of attention (64–66), that are also relevant for successfully performing the response-based Simon conflict (67, 68). Therefore, hyperactivation in AUD compared to ADHD might also be seen as a compensatory mechanism. However, we cannot derive a conclusive interpretation since the results might originate from hyperactivation in individuals with AUD or hypoactivation in ADHD. Hence, bearing our results and null findings on a neural or behavioral level in mind, we cannot ultimately determine compensatory strategies regarding interference inhibition. It should also be noted that some studies apply interference inhibition task that are combined with salient substance-related stimuli. The interaction of both, task and stimulus, leads to more pronounced results (52).

*Action withholding* elicited strong group differences between AUD and ADHD—on a behavioral and neural level. While exhibiting significantly fewer commission errors than individuals with ADHD, AUD showed strong hyperactivation in the left inferior frontal gyrus compared to ADHD, but also widespread hyperactivation in further subcortical and cortical regions similar to the comparison of AUD and HC. The left inferior frontal gyrus, overlapping with the insula, is relevant for a successful response inhibition, especially regarding the suppression of a prepotent response (69), which is necessary for successfully performing the Go-/No-go task. The striatum, however, was reported earlier as being relevant for stopping an ongoing response (63) instead of withholding a prepotent one. Most likely, we observed compensatory neural mechanisms in AUD (leading to results on the behavioral level that are comparable to HC) and ADHD (as an attempt to maintain task performance), the first group being more successful in doing so.

Regarding *action cancellation*, no subcortical differences were observed when contrasting AUD and ADHD. The typical involvement of striatal regions (63) did not become apparent in our sample. Therefore, compensatory strategies include neither subcortical hyperactivation in individuals with AUD nor the recruitment of visual or attention related regions in individuals with ADHD. Results of a direct contrast of both patient groups are most likely due to the strong hypoactivation of the TPJ in individuals with ADHD, which was also observable when contrasting ADHD to HC. Impairments in the functioning of the TPJ can be related to deficits in attention and inhibition (37, 55–57). However, one might interpret hypoactivation of motor regions in individuals with AUD as a compensatory mechanism, leading to differences in stop-signal reaction times between AUD and ADHD.

Additionally, when contrasting subprocesses and groups, individuals with AUD showed a hyperactivation during action cancellation compared to interference inhibition of the caudate, a part of the striatum. All other comparisons of subprocess and groups revealed hypoactivation within patients compared to healthy controls. Specific neural activation profiles became became apparent, indicating alterations within frontal and cingulate regions as well as parietal and temporal regions for ADHD and in parietal and motor regions as well as temporal inferior frontal and insular regions for AUD during later (action cancellation) compared to earlier processes (interference inhibition and action withholding). These group- and subprocess-specific profiles might indicate deficits in regions relevant for attention and cognitive control, as discussed above. Additional research, also due to here present study limitations, is needed to further elaborate and characterize these profiles.

### Limitations

Even though all participants were carefully selected, few reported medication or current cannabis intake only afterwards, which led to exclusion from subsequent analyses. Furthermore, some participants did not return the completed questionnaires. In addition, few participants were not able to complete the fMRI experiment or showed heavy movement in the scanner and had to be excluded from the analysis. Taken together, this resulted in a reduced number of participants in the analysis. However, neural findings can still be discussed in case of small sample sizes (70). The small sample size might also lead to null findings, since severity of either ADHD or SUD can influence neural deficits (71). In individuals with ADHD, for example, significant group differences on the behavioral level became apparent only after increasing the number of trials within the experiment [344 to 688 trials; (47)]. Due to some participants not reporting on their smoking status, we did not include this information in the analyses since this would have further reduced the sample size. However, it has been observed previously that smoking might be a potential confounder in neuroimaging studies (72, 73). The difference in age between individuals with AUD and ADHD originated from the available patients at the corresponding clinics. Regarding the effects of age on neural correlates of executive functioning in healthy individuals, older individuals showed more activation in dorsolateral, right rostolateral prefrontal and left supplementary motor regions as well as the right middle frontal gyrus (74). Even though age was included as a covariate in all analyses, the well-known influence of aging (75) remained as a limitation in this study. In addition, the amount of alcohol consumed during the last weeks was not assessed in this study. The effect of alcohol on deficits in response inhibition is only of moderate size and can be influenced by dependence severity and amount of alcohol intake (48). The combined task to assess subprocesses of response inhibition is a strength, but also a limitation to our study. A different cognitive load during the completion of the combined task, compared to separate tasks as used by others might influence the results and complicate the comparability to previous studies using separate tasks. It has been observed previously that results may vary depending on the specific task design—even though one administered the “same” inhibition task (30). When integrating our results into previous literature, one has to bear in mind that most reviews or meta-analyses combine different response inhibition tasks or stimulus modalities [e.g., (29, 36, 37, 43)]. Representing a higher ecological validity, tasks combining inhibition paradigms with alcohol-related cues (39, 52, 58, 60) might also lead to diverging findings. Furthermore, numerous studies in individuals with AUD do not assess ADHD. Adding to that, administering questionnaires only might lead to an underreporting of ADHD symptoms (76). This might impair the generalizability of those studies. The high rate of further comorbidities in AUD and ADHD (77–79) could also complicate the assessment of biological factors regarding clinical diagnosis, indicating that a transdiagnostic approach regarding impaired inhibitory control might be more promising.

### Future Directions

ADHD in combination with AUD may aggravate inhibitory deficits. Causal explanations of the relation of ADHD and AUD or preclinical impairments in response inhibition preceding or influencing higher rates of comorbidity in AUD and ADHD still have to be addressed. Further studies need to examine comorbid individuals not only including a larger sample and more trials per run in a task—but also following a longitudinal design, assessing current ADHD or markers of impulsivity. Additionally, a replication of the present study in a larger sample is highly recommended.

In case of comorbidity, treating both AUD and ADHD with a multimodal treatment approach is probably beneficial in addiction therapy, and recommended according to current guidelines, e.g., using CBT and psychopharmacological medication. For ADHD in patients with SUD, long-acting stimulants, atomoxetine, or extended release guanfacine are recommended (80, 81). CBT following inhibitory control training may lead to improved response inhibition (82, 83), not only ameliorating daily tasks, but enhancing cognitive control over substance craving and inhibitory control over impulsive substance consumption behavior. Including pharmacological treatments addressing both substance craving and response inhibition, may further be beneficial for maintaining abstinence, since individuals with a comorbid AUD and ADHD showed greater impairments during interference inhibition when being confronted with alcoholic cues (52). Cortico-striatal regions are often mentioned in relation to response inhibition in individuals with AUD and ADHD (25). It is worth noting that these regions are also involved in stress and reward processing (84, 85), and often show alterations that are associated with AUD (86). Guanfacine, for example, has been shown to have an effect on stress coping and drug craving (87), and is approved for treatment of ADHD. ADHD medication could be examined also regarding its direct effect on response inhibition in general. Since individuals with AUD or ADHD show impaired response inhibition, and stimulant treatment of ADHD in children might help to prevent later SUDs (88), a stimulant medication could be beneficial for those individuals with AUD or ADHD to improve inhibitory control. Not only should a treatment of AUD or ADHD *per se* be considered, but also the inhibitory deficits through medication or behavioral training should be addressed specifically. It has been previously shown for atomoxetine that such interventions may reduce alcohol consumption and extend the time of abstinence (48).

With respect to our behavioral results, it is still open to conclude that deficits in response inhibition due to ADHD may partially explain the high comorbidity of ADHD and AUD. However, our findings indicate that later components of response inhibition should be assessed (e.g., by using a Stop-Signal task) when examining consequences of impaired response inhibition or administering preclinical screening measures. It was observed in young heavy drinkers that only deficits in action cancellation were related to an increase in binge drinking in females (49). While interference inhibition did not result in significant observations when comparing clinical populations to healthy controls, directly contrasting ADHD and AUD led to significant observations in our sample. However, future studies should still include a healthy control group since directly contrasting AUD and ADHD leave the question of hypo- or hyperactivation open. The before mentioned studies that combined inhibition tasks with alcohol-related stimuli might not only present a limitation regarding the comparability of studies, but also a major opportunity. Following studies could focus on the more ecologically valid combination of both stimulus content and response inhibition, with respect to the influence of the environment on different subprocesses of response inhibition. Examining the influence of alcohol related stimuli at each stage (interference inhibition, action withholding and action cancellation) might further contribute to the development of interventions addressing inhibitory control in both individuals with both AUD and ADHD. In addition, the aspect of motivation during task performance needs to be taken into account. Not only does it play a role regarding drinking behavior, e.g., restraining from alcohol (82), motivation further modulates behavioral and neural findings during response inhibition paradigms (89).

## Conclusion

Neural differences were observed with respect to both the subtask of the neuroimaging paradigm and the examined groups. We observed differential neural activation in subprocesses of response inhibition when comparing individuals with ADHD and AUD patients, and healthy controls. While no effects have been shown in interference inhibition, motor inhibition seems to be more fitting for the characterization of functional neural deficits in ADHD and AUD patients. Our experimental design also revealed distinguishable neural activation patterns for individuals with ADHD and AUD even though impaired response inhibition is seen as a common phenotype of ADHD and AUD. Over all subprocesses, individuals with ADHD consistently showed hypoactivation, indicating compensatory and deficient alterations in neural subcomponents of response inhibition. Individuals with AUD, however, showed hyperactivation in earlier and hypoactivation in later subprocesses of response inhibition, suggesting that different neural subprocesses lead to similar impulse control disorders in the two patient groups. Keeping the limitation of the small sample size in mind, our results might indicate rather attention related alterations in individuals with ADHD and rather motor control related alterations in individuals with AUD compared to HC, respectively. This is relevant for the diagnosis and, even more important, for the treatment of the patients. Based on our findings, further studies should characterize these specific effects in these patient groups in more detail while also examining a larger sample.

## Data Availability Statement

The data that support the findings of this study are not publicly available due to containing information that would compromise research participant consent. We will provided data upon direct request by research colleagues following current data protection guidelines.

## Ethics Statement

The studies involving human participants were reviewed and approved by Ethics Committee of the University of Heidelberg, Germany (Study Approval Number: 2013-530N-MA). The patients/participants provided their written informed consent to participate in this study.

## Author Contributions

SG: investigation, data curation, formal analysis, writing-original draft preparation, and visualization. ML: conceptualization, supervision, project administration, and writing (review and editing). JMB: writing (review and editing). ES: conceptualization, project administration, and writing (review and editing). FM: writing (review and editing). FK: conceptualization, resources, funding acquisition, and project administration. SV-K: conceptualization, resources, project administration, funding acquisition, data curation, formal analysis, and writing (review and editing). All authors revised the manuscript critically for important intellectual content and approved the final version.

## Funding

This project was supported in part by grants from the Deutsche Forschungsgemeinschaft (TRR 265 Project ID-402170461, Project ID-421888313, Project-ID 437718741, and GRK2350-1 Project-ID 324164820).

## Conflict of Interest

ML has received honoraria as a speaker and for participation in advisory boards from MEDICE/Arzneimittel Pütter GmbH and Shire/Takeda, and travel costs from Shire/Takeda. ES has received speaker's honoraria from Shire and MEDICE/Arzneimittel Pütter GmbH. FM has received speaker's honoraria from Janssen. The remaining authors declare that the research was conducted in the absence of any commercial or financial relationships that could be construed as a potential conflict of interest.

## Publisher's Note

All claims expressed in this article are solely those of the authors and do not necessarily represent those of their affiliated organizations, or those of the publisher, the editors and the reviewers. Any product that may be evaluated in this article, or claim that may be made by its manufacturer, is not guaranteed or endorsed by the publisher.
