# Supplementary material for: Stop What You're Doing!—An fMRI Study on Comparisons of Neural Subprocesses of Response Inhibition in ADHD and Alcohol Use Disorder
Source: Front Psychiatry. 2021 Sep 16;12:691930. doi: 10.3389/fpsyt.2021.691930 (PMC8481878; doi:10.3389/fpsyt.2021.691930)
Supplement: Supplementary file 1 [file Data_Sheet_1.pdf]

Stop what you're doing! - An fMRI Study on Comparisons of Neural Subprocesses of Response Inhibition in ADHD and Alcohol Use Disorder

Supplementary Material

1. Questionnaires

The questionnaire-based screening for AUD and AHD included the following: Alcohol Use Disorder Identification Test (AUDIT; Reinert and Allen, 2002), cut-off < 8 for HC and ADHD; Wender-Utah-Rating-Scale (WURS-k; Retz-Junginger et al., 2002), cut-off < 30 for HC and AUD; ADHD self-report scale (ADHD-SR; Rösler et al., 2008), cut-off < 6 for items one to nine and cut-off < 6 for items 10 to 18; Adult ADHD Self-Report Scale (ASRS-SR; Kessler et al., 2005), cut-off < 14 for HC and AUD. After a successful group allocation and study inclusion, all participants filled out the Alcohol Dependence Scale (ADS; Skinner and Horn, 1984), the AUDIT (Reinert and Allen, 2002), the Alcohol Urge Questionnaire (AUQ; Bohn et al., 1995), the WURS-k (Retz-Junginger et al., 2002), the ADHD-SR (Rösler et al., 2008), and the Fagerström Test for Nicotine Dependence (FTND; Heatherton et al., 1991).

2. Hybrid Response Inhibition task

Before performing the Hybrid Response Inhibition (HRI) functional resonance imaging (fMRI) task (Sebastian et al., 2013) in the scanner, participants received a short training run on a laptop computer outside the scanner. During the fMRI experiment, three runs of the HRI task were performed with a short break in between. Each run had a duration of 8:48 minutes. Participants were instructed to respond as quickly and accurately as possible prior to each run. Each run stated with a visual presentation of the instruction for 5000 ms, followed by a fixation cross in the center of the screen for further 500 ms. During the 160 trials of each run, four different stimuli conditions were presented in a pseudo-randomized order: a congruent go condition (62.5%; a left pointing arrow in the left hemisphere of the ellipse and vice versa), an incongruent go condition (12.5%; a left pointing arrow in the right hemisphere of the ellipse and vice versa), a no-go condition (12.5%; a congruent condition with a change of white to blue of the ellipse at the onset of the arrow), and a stop condition (12.5%; a congruent condition with a change of white to blue of the ellipse after a delay). The variable stop-signal delay (SSD) in the stop condition was adapted to the individual performance. A probability of 50% of correct inhibitions per run was achieved via a staircase procedure. After a commission error (response was not inhibited) the initial SSD of 220 ms was decreased by 50 ms (minimum SSD = 20 ms). A correct stop was followed by an increase of the SSD by 50 ms. Moreover, the length of the inter-stimulus-interval was jittered by 1500 ms (SD 372 ms). Each trial had the following procedure: a white ellipse circled the fixation cross for 500 ms followed by the appearance of a white arrow (for 1000 ms or until a button was pressed) either on the right or left side of the cross within the ellipse. Individuals had to respond by pressing the corresponding button (left for an arrow pointing to the left and vice versa). Additionally, they had to withhold their reaction whenever the ellipse turned blue (see Figure 1 of the main manuscript). Participants were asked to fixate on the cross that appeared between trials throughout the task.

3. Sample Size Calculation and Post-hoc Analyses

Sample size calculations using the software G*power (http://www.gpower.hhu.de) resulted in N = 22 individuals per group. This estimate is sufficient to detect a large effect with a power above 80% and an alpha error probability

of 0.05 for our analyses (ANOVA, three groups). Post-hoc analyses (effect sizes and power estimations) regarding neural findings of subprocesses of response inhibition were conducted using SPM, SPSS, and the software g*power (Faul et al., 2007). Firstly, brain region masks were created using the WFU_Pick_Atlas (anatomical automatic labeling) implemented in SPM. Then, eigenvariates were extracted from the first-level analyses of both HC and the group comparisons within these masks. For the analyses of the healthy controls only, we used the bilateral supplementary motor area (interference inhibition), bilateral fusiform gyrus (action withholding) and bilateral insula (action cancellation) as masks. Regarding group comparisons, we used the right precuneus (interference inhibition), the left pars orbitalis of the inferior frontal gyrus, bilateral caudate and thalamus, right angular and supramarginal gyri (action withholding), and the right angular and supramarginal gyri and right pre- and postcentral gyri (action cancellation) as masks. Effect sizes (following Cohen's *d*) and power of the fMRI analyses were then calculated. Results are reported below each table of brain regions (tables A.3 – A.6).

4. Head motion – calculation and group comparison

During the fMRI analysis procedure, head motion was estimated both regarding translation and rotation (3 parameters each: xyz). All six motion regressors that were estimated during the preprocessing of the fMRI data were extracted. The mean per regressor and participant was calculated. Additionally, total displacement and scan-to-scan displacement can be determined to describe the absolute and relative motion, respectively (Wilke, 2014, Wilke, 2012). To do so, the 'motion fingerprint' extension for SPM was used (http://www.medizin.uni-tuebingen.de/kinder/en/research/ neuroimaging/software/). Using ANOVA, group differences regarding all six motion regressors, the total displacement, and the scan-to-scan displacement were examined. No significant group differences were observed regarding all eight measures. Results from these analyses are reported in table A.3.

1. CONSORT flow-chart

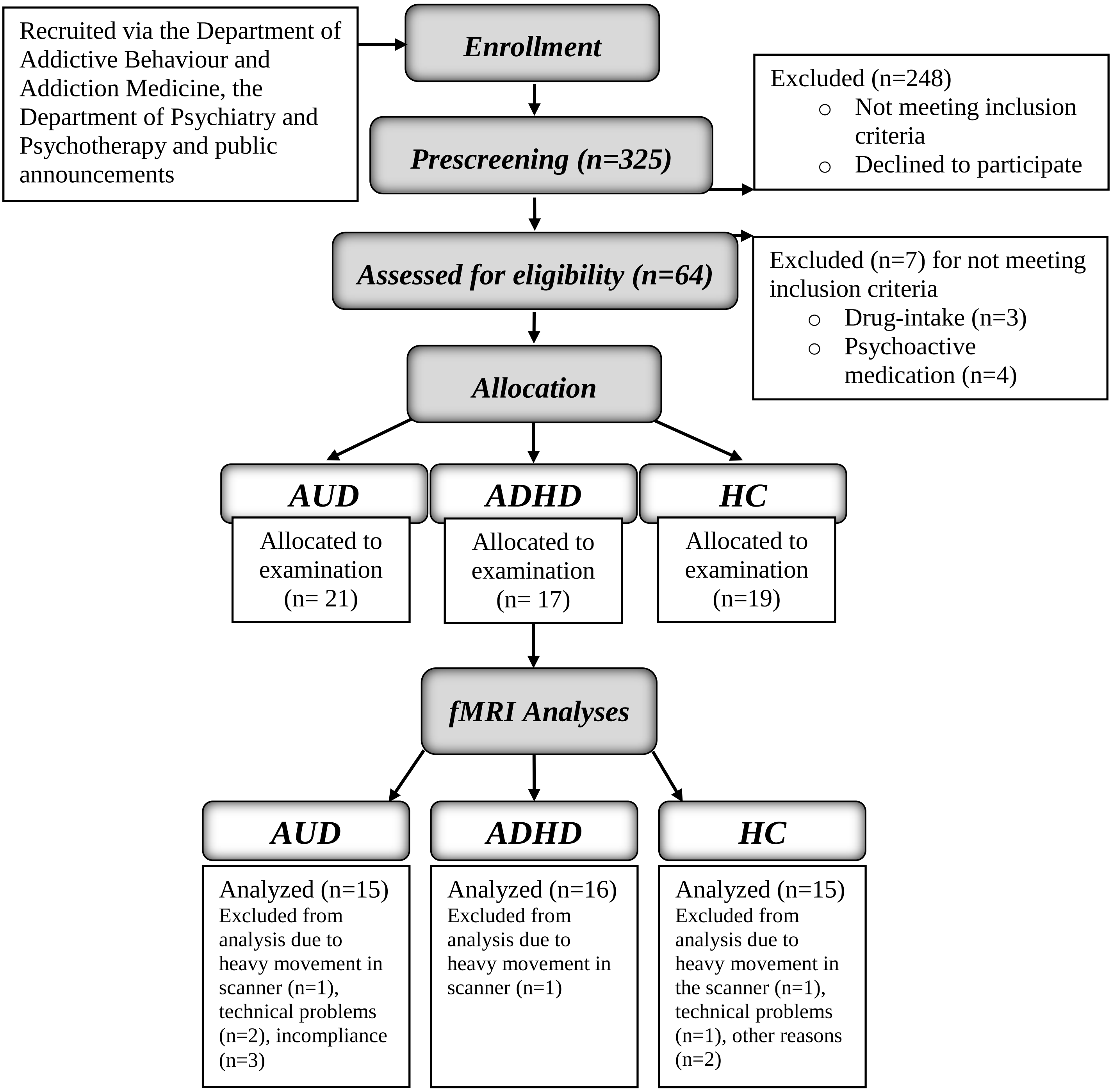

Supplementary tables

Table A.1: Inclusion and exclusion criteria

Inclusion criteria	Exclusion criteria
Man or woman	Severe physical illness, neurological disorders, history of brain injury
Age between 18 and 65 years	Current Axis I psychiatric diagnoses other than AUD or ADHD
Signed written informed consent following the Declaration of Helsinki	Severe withdrawal symptoms (CIWA-Ar > 4; Sullivan et al. 1989) at the time of MRI investigation
Normal or corrected to normal vision	Cocaine, amphetamine, opiate dependence lifetime
Abstinence from alcohol (3-6 weeks)	Intake of drugs (other than alcohol or tobacco) within the last 3 weeks
ADHD according to current clinical guidelines	Psychotropic medication within the last 3 days or therapy with methylphenidate within the last 8 weeks
Alcohol Use Disorder according to ICD -10	Common exclusion criteria for MRI (e.g. metal implants, claustrophobia, pregnancy)

Table A.2: Behavioral data of patients (AUD, ADHD) vs. controls for the HRI-task.

	**Patients (AUD+ADHD) (*N* = 31)**	**HC (*N* = 15)**	**Two sample t-Test (2-tailed)**
**Reaction time congruent trials** [ms; mean±SD]	521±111	507±105	t(44) = 0.65, p = .518
**Reaction time incongruent trials** [ms; mean±SD]	618±109	609±100	t(44) = 0.27, p = .798
**Commission errors (no-go)** [%; mean±SD]	6.0±11.3	3.6±4.6	t(44) = 0.79, p = .434
**Omission errors (no-go)** [%; mean±SD]	3.0±5.1	6.8±16.6	t(15.3) = -0.87, p = .398
**Failure to stop** [%; mean±SD]	45.2±12.8	42.3±13.0	t(44) = 0.71, p = .480
**Interference effect** [ms; mean±SD]	97±65	101±48	t(44) = -0.22, p = .829
**Stop-signal reaction time** [ms; mean±SD]	269±61	228±64	**t(44) = 2.11, p = .040**

Table A.3: Motion parameters of AUD, ADHD, and HC during the HRI-task.

	**AUD (*N* = 15)**	**ADHD (*N* = 16)**	**HC (*N* = 15)**	**ANOVA**
**Total displacement** [mean±SD]	0.63±0.35	0.73±0.38	0.54±0.25	F(2,43) = 1.25, p = .295
**Scan-to-scan displacement** [mean±SD]	0.10±0.05	0.11±0.06	0.09±0.03	F(2,43) = 0.52, p = .594

Note**:** ANOVA= one-way analysis of variance. No significant group differences (ANOVA, p > 0.05) were observed regarding the 6 motion parameters (xyz translation and xyz rotation).

Table A.4: Brain activation during interference inhibition, action withholding and action cancellation in healthy controls. CDT of P < 0.01 (k ≥ 452), N = 15.

Side	Lobe	Brain Areas	Brodmann Area	Cluster Size	MNI Coordinates x	y	z	t_{max}
**Interference inhibition**								
Right	Parietal lobe	Inferior, superior parietal lobule, supramarginal gyrus, precuneus, postcentral gyrus	2,7,19,37,40	12,1589	-34	-50	44	12.16
Left	Parietal lobe	Inferior, superior parietal lobule, supramarginal gyrus, precuneus, postcentral gyrus						
Left	Temporal lobe	Middle, inferior temporal gyrus, fusiform gyrus						
Right	Occipital lobe	Middle, superior occipital gyrus						
Left	Occipital lobe	Inferior, middle, superior occipital gyrus						
Left	Frontal lobe	Superior, middle, medial frontal gyrus, precentral gyrus, supplementary motor area	6	2,314	-30	0	58	8.72
Right	Frontal lobe	Supplementary motor area						
Right	Temporal lobe	Middle, inferior temporal gyrus, fusiform gyrus	37,19	2,187	34	-48	-30	8.51
Right	Occipital lobe<br>Cerebellum	Inferior occipital gyrus						
Right	Frontal lobe	Superior, middle frontal gyrus, precentral gyrus	6	746	36	-4	56	6.31
Left	Frontal lobe	Inferior frontal gyrus, precentral gyrus	9,6	494	-50	6	30	6.02
**Action withholding**								
Right	Parietal lobe	Superior parietal lobule, angular gyrus	7,18,19,37	15.,148	30	-78	-10	15.51
Right	Temporal lobe	Middle, inferior temporal gyrus, fusiform gyrus						
Left	Temporal lobe	Inferior temporal gyrus, fusiform gyrus						
Right	Occipital lobe	Inferior, middle, superior occipital gyrus, lingual gyrus, calcarine, cuneus						
Left	Occipital lobe	Inferior, middle, superior occipital gyrus, lingual gyrus, calcarine, cuneus						
**Action cancellation**								
Right	Parietal lobe	Inferior, superior parietal lobule, supramarginal gyrus, angular gyrus, precuneus	7,18,19,21,22, 37,40	11,692	40	-86	10	9.97
Right	Temporal lobe	Middle, inferior, superior temporal gyrus, fusiform gyrus						
Right	Occipital lobe	Inferior, middle, superior occipital gyrus, lingual gyrus, calcarine, cuneus						
Right	Frontal lobe	Superior, middle, medial, inferior frontal gyrus, precentral gyrus, supplementary motor area	6,8,9,47	4,476	32	20	-2	7.82
Let	Frontal lobe	Supplementary motor area						
Right	Temporal lobe	Temporal pole						
Right		Insula, putamen, amygdala						
Left	Frontal lobe	Inferior frontal gyrus, frontal pole	13,38,47	1,336	-32	18	-16	7.82
Left		Insula, putamen						
Left	Temporal lobe	Inferior temporal gyrus, fusiform gyrus	7,18,19,37	5,280	-22	-98	24	7.75
Left	Occipital lobe	Superior, middle, inferior occipital gyrus, lingual gyrus, cuneus						
Left	Parietal lobe	Superior, inferior parietal lobule, angular gyrus, supramarginal gyrus, precuneus	7,40	1,563	-54	-48	32	5.26

Note: Effect size and power were calculated for the following regions: interference inhibition: bilateral supplementary motor (effect size d = 0.827, power = 0.845); action withholding: bilateral fusiform gyrus (effect size d = 1.133, power = 0.983); action cancellation: bilateral insula (effect size d = 0.958, power = 0.931).

Table A.5: Brain activation during interference inhibition. CDT of P < 0.01 (k ≥ 460).

Side	Lobe	Brain Areas	Brodmann Area	Cluster Size	MNI Coordinates x	y	z	t_{max}
**AUD > ADHD**								
Right	Parietal lobe	Superior parietal lobule, precuneus	7,19	701	10	-68	54	3.78
Right	Occipital lobe	Superior, middle occipital gyrus						

Note: Effect size and power were calculated for the following region: AUD > ADHD: right precuneus (effect size *d* = 1.674, power = 0.994).

Table A.6: Brain activation during action withholding. CDT of P < 0.01 (k ≥ 460).

Side	Lobe	Brain Areas	Brodmann Area	Cluster Size	MNI Coordinates x	y	z	t_{max}
**AUD > ADHD**								
Left	Frontal lobe	Inferior, middle frontal gyrus	47	1,026	-24	36	-10	5.14
Left		Insula						
Left	Parietal lobe	Inferior parietal lobule, angular gyrus	19	681	-46	-54	32	4.46
Left	Occipital lobe	Middle occipital gyrus						
Right	Temporal lobe	Fusiform gyrus		525	32	-26	-10	4.24
Right		Hippocampus, parahippocampal gyrus						
Left	Temporal lobe	Fusiform gyrus		1,544	-20	-8	24	4.23
Left		Thalamus, hippocampus, parahippocampal gyrus, caudate						
Right	Frontal lobe	Medial frontal gyrus	32	757	2	26	-12	4.11
Left	Frontal lobe	Medial frontal gyrus						
Right		Cingulate gyrus (anterior)						
Left		Cingulate gyrus (anterior)						
Left	Frontal lobe	Superior, medial frontal gyrus	6,8	629	-6	30	34	4.09
Right		Thalamus, caudate		572	12	-20	-18	3.97
Right	Parietal lobe	Precuneus	7,31	1,379	0	-40	30	3.73
Left	Parietal lobe	Precuneus						
Right		Cingulate gyrus (middle, posterior)						
Left		Cingulate gyrus (middle, posterior)						
**AUD > HC**								
Left		Thalamus, Caudate,		1,115	-10	-10	16	5.14
Left	Temporal lobe	Temporal pole	38	805	-4	12	-26	4.98
Left	Frontal lobe	Middle, inferior frontal gyrus	47	1,200	-28	34	-12	4.65
Left	Frontal lobe	Medial frontal gyrus						
Left		Insula						
Right		Caudate, putamen, thalamus, pallidum, insula		1,042	22	-10	4	4.45
Left	Parietal lobe	Inferior parietal lobule, angular gyrus	39,40	859	-38	72	44	4.04
Left	Occipital lobe	Middle occipital gyrus						
Left	Temporal lobe	Superior, middle temporal gyrus	21	465	-54	-8	-28	4.01
Right	Temporal lobe	Superior temporal gyrus	13	470	44	-22	2	3.95
Right		Hippocampus, insula						
Right	Parietal lobe	Precuneus	7	654	-6	-72	30	3.76
Left	Parietal lobe	Precuneus						
Left	Occipital lob	Cuneus						
**ADHD < HC**								
Right	Parietal lobe	Angular gyrus, supramarginal gyrus	31	865	22	-44	32	4.59
Right	Limbic lobe	Cingulate gyrus (middle, posterior)						
Left	Limbic lobe	Cingulate gyrus (middle, posterior)						

Note: Effect size and power were calculated for the following regions: AUD > ADHD: left inferior frontal gyrus (p. orbitalis) (effect size d = 1.415, power = 0.967); AUD > HC: bilateral caudate and thalamus (effect size d = 1.688, power = 0.994); ADHD < HC: right angular and supramarginal gyri (effect size d = 0.909, power = 0.686).

Table A.7: Brain activation during action cancellation. CDT of P < 0.01 (k ≥ 460).

Side	Lobe	Brain Areas	Brodmann Area	Cluster Size	MNI Coordinates x	y	z	t_{max}
**AUD > ADHD**								
Right	Cerebellum			464	56	-64	-40	4.10
Right	Parietal lobe	Angular gyrus, supramarginal gyrus	40	574	58	-50	24	3.71
Right	Temporal lobe	Middle, superior temporal gyrus						
Left	Temporal lobe	Fusiform gyrus	18,19	968	-8	-64	-4	3.64
Left	Occipital lobe	Inferior occipital gyrus, lingual gyrus						
**AUD < HC**								
Right	Frontal lobe	Superior frontal gyrus, middle frontal gyrus, precentral gyrus	3,4,6	1,658	48	0	40	3.71
Right	Parietal lobe	Postcentral gyrus						
Left	Frontal lobe	Precentral gyrus	2,3,6,40	1,483	-60	-28	26	3.55
Left	Parietal lobe	Inferior parietal lobule, postcentral gyrus, supramarginal gyrus						
Left	Temporal lobe	Superior temporal gyrus						
**ADHD < HC**								
Right	Parietal lobe	Angular gyrus, supramarginal gyrus	40	643	46	-48	28	4.42
Right	Temporal lobe	Superior temporal gyrus						
Right	Temporal lobe	Inferior temporal gyrus, fusiform gyrus	19,37	1,530	44	-56	-20	4.01
Right	Occipital lobe	Inferior occipital gyrus, lingual gyrus						
Left	Temporal lobe	Fusiform gyrus	19	660	-24	-90	20	3.58
Left	Occipital lobe	Inferior, middle, superior occipital gyrus						
Left	Parietal lobe	Precuneus	7,31	471	-6	-54	46	3.27
Right		Cingulate gyrus (middle)						
Left		Cingulate gyrus (middle)						

Note: Effect size and power were calculated for the following regions: AUD > ADHD: right angular and supramarginal gyri (effect size d = 0.836, power = 0.614); AUD < HC: right pre- and postcentral gyri (effect size d = 1.054, power = 0.796); ADHD < HC: right angular and supramarginal gyrus (effect size d = 1.006, power = 0.772).

Table A.8: Brain activation during action withholding > interference inhibition. CDT of P < 0.01 (k ≥ 460).

Side	Lobe	Brain Areas	Brodmann Area	Cluster Size	MNI Coordinates x	y	z	t_{max}
**HC > ADHD**								
Right	Parietal lobe	Angular gyrus, parietal inferior gyrus	39,40	890	44	-52	32	3.94
**HC < AUD**								
Right		Caudate		531	28	20	28	3.78

Table A.9: Brain activation during action cancellation > interference inhibition. CDT of P < 0.01 (k ≥ 460).

Side	Lobe	Brain Areas	Brodmann Area	Cluster Size	MNI Coordinates x	y	z	t_{max}
**HC > ADHD**								
Left	Frontal lobe	Superior frontal gyrus	9,10,32	2,392	-8	28	36	4.14
Right	Frontal lobe	Superior frontal gyrus						
Left		Anterior, middle cingulate gyrus						
Right		Anterior, middle cingulate gyrus						
Right	Parietal lobe	Angular, supramarginal gyrus, inferior parietal gyrus	39,40	952	52	-64	50	4.41
**HC > AUD**								
Left	Parietal lobe	Inferior parietal lobule, postcentral gyrus, supramarginal gyrus	2,40	1,321	-62	-28	26	4.16
Left	Temporal lobe	Superior temporal gyrus						
Right	Parietal lobe	Postcentral gyrus, supramarginal gyrus	3,4,6,24	2,970	44	-14	56	4.45
Right	Frontal lobe	Middle, inferior frontal gyrus, precentral gyrus, supplementary motor area						
Left	Frontal lobe	Supplementary motor area						
Right		Middle cingulate gyrus						

Table A.10: Brain activation during action cancellation > action withholding. CDT of P < 0.01 (k ≥ 460).

Side	Lobe	Brain Areas	Brodmann Area	Cluster Size	MNI Coordinates x	y	z	t_{max}
**HC > ADHD**								
Right	Occipital lobe	Superior, middle occipital gyrus, lingual gyrus, calcarine	18,19,22	3,472	14	-64	0	4.28
Left	Occipital gyrus	Calcarine						
Right	Temporal lobe	Superior, middle, inferior temporal gyrus, fusiform gyrus						
Right	Parietal lobule	Supramarginal gyrus						
Left	Occipital lobe	Superior, middle occipital gyrus,	18,19	750	-56	-80	6	4.20
Left	Temporal lobe	Middle temporal gyrus						
Left	Parietal lobe	Superior, inferior parietal gyrus, precuneus	7,40	940	-60	-56	46	3.48
Left		Middle cingulate gyrus						
**HC > AUD**								
Left	Parietal lobe	Inferior parietal lobule, postcentral gyrus, supramarginal gyrus	2,40	1,206	-56	-32	36	4.21
Left	Temporal lobe	Superior temporal gyrus						
Right	Temporal lobe	Superior temporal gyrus	22	698	42	-22	4	4.19
Right		Insula						
Left	Frontal lobe	Middle, inferior frontal gyrus	9,45	514	-38	26	36	4.03
Left	Temporal lobe	Temporal pole	38,47	475	-34	18	-18	3.87
Left	Frontal lobe	Inferior frontal gyrus						
Left		Insula						

Table A.11: Brain activation during action withholding in positive relation to commission errors. CDT of P < 0.01 (k ≥ 460).

**Side**	**Lobe**	**Brain Areas**	**Brodmann Area**	**Cluster Size**	**MNI Coordinates**			**t_{max}**
					**x**	**y**	**z**	
Left	Parietal lobe	Superior, inferior parietal lobule, precuneus, postcentral gyrus	2,6,40	6,979	32	-42	70	6.06
Right	Parietel lobe	Inferior parietal lobule, supramarginal gyrus, postcentral gyrus						
Left	Frontal lobe	Superior frontal gyrus, precentral gyrus, supplementary motor area						
Right	Frontal lobe	Superior frontal gyrus, precentral gyrus, supplementary motor area						
Left	Occipital lobe	Middle occipital gyrus, cuneus, lingual gyrus	17,18	3,114	22	-90	12	6.01
Right	Occipital lobe	Superior, middle, inferior occipital gyrus, cuneus, calcarine						
Left	Frontal lobe	Middle frontal gyrus	11	694	-22	42	6	5.38
Left		Insula						
Right	Temporal lobe	Middle, inferior temporal gyrus	22	678	74	-44	4	5.20
Left	Frontal lobe	Inferior frontal gyrus (pars opercularis)	13	1,395	-24	-6	0	4.95
Left		Insula, pallidum, putamen						
Right		Pallidum, caudate						
Right	Frontal lobe	Middle, inferior frontal gyrus	10	753	40	28	26	4.33
Left	Temporal lobe	Middle temporal gyrus	22	807	-42	-30	-4	3.93
Left		Hippocampus						

Note: All individuals (N = 46) were included in this regression model. A positive correlation between neural activation during action withholding with the rate of commission errors was observed. No negative correlation was observed.

Supplementary Figures

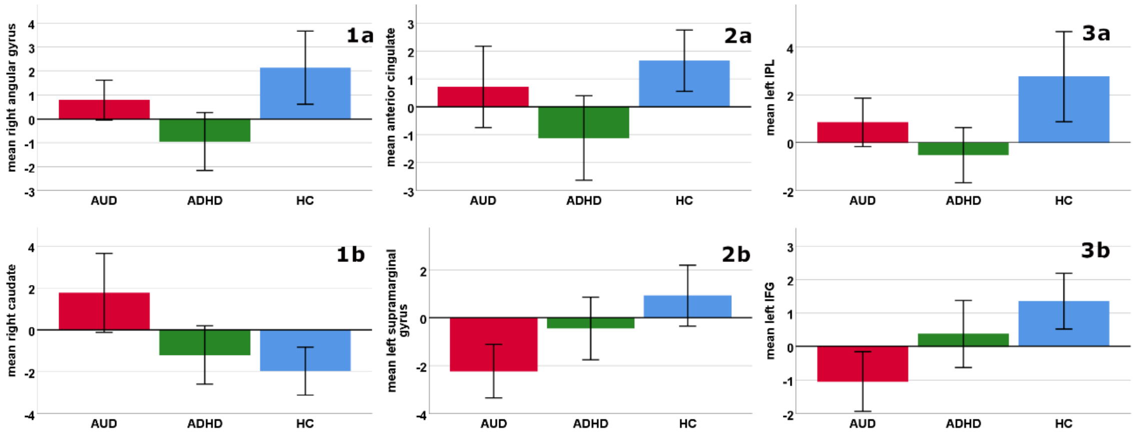

Figure A.1: **Group comparisons (AUD, ADHD, HC) regarding differences in subcomponents of response inhibition. Mean of activation regarding specific, significant task comparisons are displayed for AUD, ADHD and HC.** Eigenvariates within anatomical regions (see also figure 4, tables A.8 - A.10) were extracted. ANOVA and corresponding post-how tests were used to confirm significant group differences ($p < 0.05$). **Left: Action withholding > interference inhibition**; 1a: HC > ADHD, right angular gyrus; 1b: HC < AUD, right caudate. **Middle: Action cancellation > interference inhibition**; 2a: HC > ADHD, anterior cingulate; 2b: HC > AUD, left supramarginal gyrus. **Right: Action cancellation > action withholding**; 3a: HC > ADHD, left inferior parietal lobule (IPL); 3b: HC > AUD, left inferior frontal gyrus (IFG). RED: individuals with AUD (N = 15); GREEN: individuals with ADHD (N = 16); BLUE: healthy individuals (N = 15). The 95-% confidence interval is displayed.

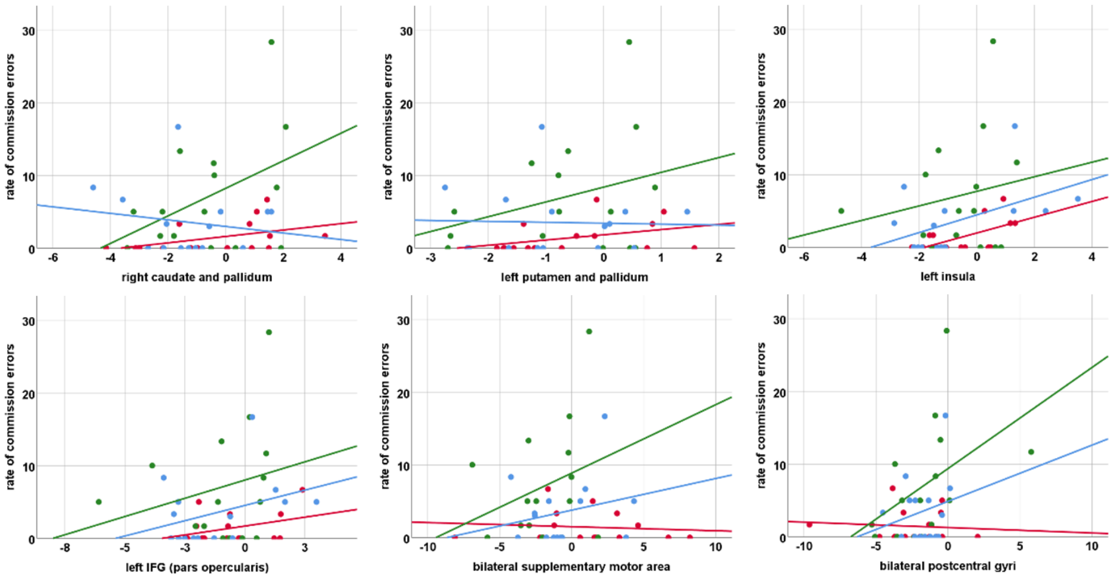

Figure A.2: Scatterplots displaying the rate of commission errors (go-/no-go; in percentage) and neural activation with respect to the first level contrast of action withholding. Individual eigenvariates were extracted within the described anatomical regions. RED: individuals with AUD (N = 15); GREEN: individuals with ADHD (N = 15); BLUE: healthy individuals (N = 15). One high potential outlier (ADHD) was excluded following the inspection of a boxplot and the computation of inter quartile ranges in SPSS.

BOHN, M. J., KRAHN, D. D. & STAEHLER, B. A. 1995. Development and initial validation of a measure of drinking urges in abstinent alcoholics. *Alcohol Clin Exp Res,* 19**,** 600-6.

FAUL, F., ERDFELDER, E., LANG, A.-G. & BUCHNER, A. 2007. G*Power 3: A flexible statistical power analysis program for the social, behavioral, and biomedical sciences. *Behavior Research Methods,* 39**,** 175-191.

HEATHERTON, T. F., KOZLOWSKI, L. T., FRECKER, R. C. & FAGERSTROM, K. O. 1991. The Fagerström test for nicotine dependence: a revision of the Fagerstrom Tolerance Questionnaire. *Addiction,* 86**,** 1119-1127.

KESSLER, R. C., ADLER, L., AMES, M., DEMLER, O., FARAONE, S., HIRIPI, E., HOWES, M. J., JIN, R., SECNIK, K., SPENCER, T., USTUN, T. B. & WALTERS, E. E. 2005. The World Health Organization Adult ADHD Self-Report Scale (ASRS): a short screening scale for use in the general population. *Psychol Med,* 35**,** 245-56.

REINERT, D. F. & ALLEN, J. P. 2002. The Alcohol Use Disorders Identification Test (AUDIT): a review of recent research. *Alcohol Clin Exp Res,* 26**,** 272-279.

RETZ-JUNGINGER, P., RETZ, W., BLOCHER, D., WEIJERS, H.-G., TROTT, G.-E., WENDER, P. H. & RÖSSLER, M. 2002. Wender Utah Rating Scale (WURS-k) Die deutsche Kurzform zur retrospektiven Erfassung des hyperkinetischen Syndroms bei Erwachsenen. *Der Nervenarzt,* 73**,** 830-838.

RÖSLER, M., RETZ, W., RETZ-JUNGINGER, P., STIEGLITZ, R., KESSLER, H., REIMHERR, F. & WENDER, P. 2008. ADHS-diagnose bei Erwachsenen. *Der Nervenarzt,* 79**,** 320-327.

SEBASTIAN, A., POHL, M. F., KLOPPEL, S., FEIGE, B., LANGE, T., STAHL, C., VOSS, A., KLAUER, K. C., LIEB, K. & TUSCHER, O. 2013. Disentangling common and specific neural subprocesses of response inhibition. *Neuroimage,* 64**,** 601-15.

SKINNER, H. A. & HORN, J. L. 1984. *Alcohol dependence scale (ADS): User's guide*, Addiction Research Foundation.

WILKE, M. 2012. An alternative approach towards assessing and accounting for individual motion in fMRI timeseries. *NeuroImage,* 59**,** 2062-2072.

WILKE, M. 2014. Isolated assessment of translation or rotation severely underestimates the effects of subject motion in fMRI data. *PloS one,* 9**,** e106498-e106498.
